# Bridge- and Solvent-Mediated Intramolecular Electronic Communications in Ubiquinone-Based Biomolecular Wires

**DOI:** 10.1038/srep10352

**Published:** 2015-05-21

**Authors:** Xiao-Yuan Liu, Wei Ma, Hao Zhou, Xiao-Ming Cao, Yi-Tao Long

**Affiliations:** 1 Key Laboratory for Advanced Materials & Department of Chemistry, East China University of Science and Technology, Shanghai 200237, P. R. China

## Abstract

Intramolecular electronic communications of molecular wires play a crucial role for developing molecular devices. In the present work, we describe different degrees of intramolecular electronic communications in the redox processes of three ubiquinone-based biomolecular wires (Bis-CoQ_0_s) evaluated by electrochemistry and Density Functional Theory (DFT) methods in different solvents. We found that the bridges linkers have a significant effect on the electronic communications between the two peripheral ubiquinone moieties and solvents effects are limited and mostly depend on the nature of solvents. The DFT calculations for the first time indicate the intensity of the electronic communications during the redox processes rely on the molecular orbital elements V_L_ for electron transfer (half of the energy splitting of the LUMO and LUMO+1), which is could be affected by the bridges linkers. The DFT calculations also demonstrates the effect of solvents on the latter two-electron transfer of Bis-CoQ_0_s is more significant than the former two electrons transfer as the observed electrochemical behaviors of three Bis-CoQ_0_s. In addition, the electrochemistry and theoretical calculations reveal the intramolecular electronic communications vary in the four-electron redox processes of three Bis-CoQ_0_s.

Molecular wires^1^,^2^, which are composed of a molecular chain promoting the electronic communication between the two groups attached to terminals of the chain, have been extensively investigated^3^,^4^,^5^,^6^,^7^,^8^,^9^,^10^,^11^ due to their promising applications including photosystem^12^,^13^,^14^,^15^ and molecular electronics^16^,^17^. However, a major challenge is to find appropriate molecules that display effective charge-transfer as widely found in nature, such as the redox cofactors in photosynthetic reaction. For example, as an essential cofactor, quinone serves as a mobile carrier for electrons and protons transfer in the bioenergetic cycle of photosynthesis to generate transmembrane proton gradients driving the synthesis of adenosine triphosphate (ATP)^18^. In photosynthesis II, quinone undergoes a two-electron, two-proton redox process to fulfill the intracellular electron transfer and transmembrane transport of protons^18^,^19^. The fine-tuned electrons transfer via these cofactors results in nearly 100% photoconversion quantum yield in photosynthesis^20^, whereas such levels of performance have never been obtained in artificial systems so far. In consideration of the crucial role in photosynthetic processes, quinone may act as an excellent terminal group to acquire effective electronic communications in quinone-based biomolecular wires and have the potential applications in artificial photosynthetic system and electronic devices.

To this end, in the present study we designed and synthesized three ubiquinone-based biomolecular wires (Bis-CoQ_0_s) (depicted in Fig. 1) coupled by different bridge linkers and studied the electronic communications between two ubiquinone groups during the four electrons redox processes using the electrochemistry and Density Functional Theory (DFT) methods in aprotic organic solvents and oxygen-free environment mimicking the nonpolar environment in living cells. In this way, we hope to fine-tune the level of electronic communications between the two peripheral ubiquinone groups and explore the molecular structures-based reasons causing the different degree electronic communications and further extend the applications of ubiquinone as biomolecular wires in artificial photosynthetic system and electronic components.

## Results

### Electrochemical studies of three Bis-CoQ_0_s

Our previous study has shown that the strong intramolecular communications lead to three-step, consecutive four-electron redox processes of Bis-CoQ_0_ 1 in aprotic solvent to generate the unstable reduced intermediate species-monoradicals (CoQ_0_-CoQ_0_^.−^), dianions (CoQ_0_^.−^-CoQ_0_^.−^) and tetraanions (CoQ_0_^2−^-CoQ_0_^2−^)^21^. As shown in Fig. 2, in the four-electron redox processes of three Bis-CoQ_0_s, Bis-CoQ_0_ 1 displays three redox steps with the formal potentials at −1.024 V, −1.210 V and −1.696 V; Bis-CoQ_0_ 2 exhibits two steps redox processes at −1.055 V and −1.739 V, coupled with an ill-defined shoulder in first reduction peak; Bis-CoQ_0_ 3 exhibits two pairs of sharp redox peaks at −1.027 V and −1.704 V, which is similar with the electrochemical property of CoQ_0_ located at −1.059 V and −1.721 V.

**Figure 1:**
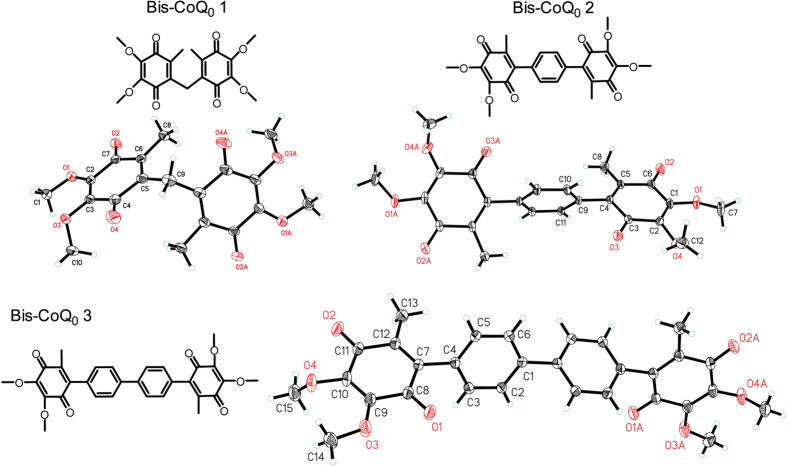
Structures of three Bis-CoQ_0_s in the research.

**Figure 2:**
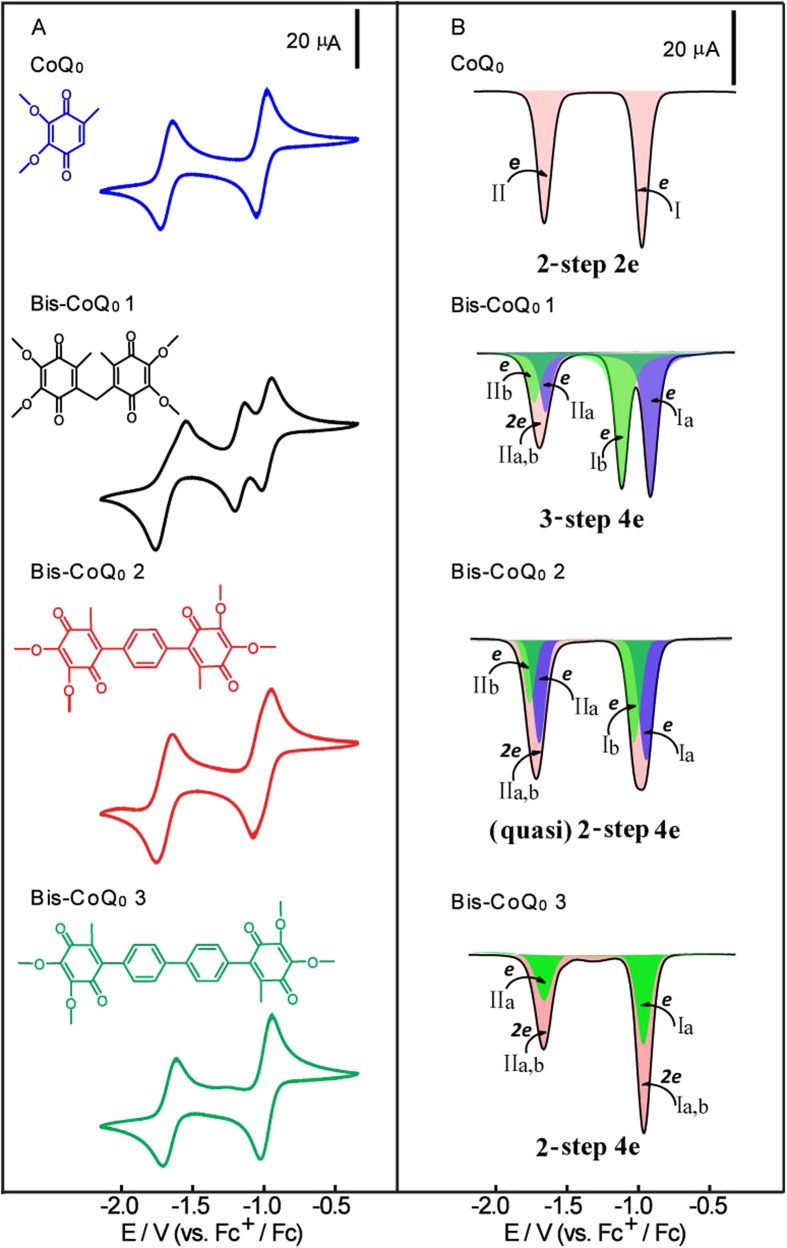
(**A**) CV curves of 1.0 mM CoQ_0_, Bis-CoQ_0_ 1, Bis-CoQ_0_ 2 and Bis-CoQ_0_ 3 obtained at a GC electrode (ø = 3 mm) in anhydrous and deoxygenated CH_3_CN containing 0.1 M TBAP at scan rate 0.100 Vs^−1^; (**B**) experimental (black line) and simulated (colored) DPV curves of 1.0 mM CoQ_0_, Bis-CoQ_0_ 1, Bis-CoQ_0_ 2 and Bis-CoQ_0_ 3, increment E = 0.004 V, frequency = 15 Hz.

The splitting of reduction peaks of first two electrons transfer processes of Bis-CoQ_0_s can be related to the electronic communications between the two peripheral ubiquinone moieties, which could be fine-tuned by the bridge linkers from the electrochemical results. The three-step four-electron redox process of Bis-CoQ_0_ 1 indicates a strong intramolecular electronic communication between two methylene-coupled quinonyl groups^21^. While, the ill-defined shoulder peak of Bis-CoQ_0_ 2 suggests that this peak contains two electron transfer processes, which do not occur simultaneously and have a time delay interval. The stepwise electron-transfer of Bis-CoQ_0_ 2 is more significant in DPV results as shown in Fig. 2B, where the full width at half maximum (FWHM) for the first two-electron reduction process of Bis-CoQ_0_ 2 is much broader than that for CoQ_0_ and Bis-CoQ_0_ 1 (Table 1). The simulated DPV curve of Bis-CoQ_0_ 2 also confirms that two close one-electron transfer steps are included in the first reduction peak. These results reveal that the intramolecular electronic communication is feeble in phenylene-linked Bis-CoQ_0_ 2. However, the similar electrochemical behaviors of Bis-CoQ_0_ 3 and CoQ_0_ demonstrate no intramolecular electronic communication in Bis-CoQ_0_ 3, making two peripheral ubiquinone groups completely independent, thus the FWHM for both reduction peaks of Bis-CoQ_0_ 3 are equal to that of CoQ_0_ (Table 1). Additionally, the peak area of first reduction process of Bis-CoQ_0_ 2 and Bis-CoQ_0_ 3 is greater than that of CoQ_0_, demonstrating the reduction processes of the both Bis-CoQ_0_s undergo a two-electron transfer step. The different degrees of intramolecular electronic communications in redox processes of three Bis-CoQ_0_s may be attributed to the increasing distance between two peripheral quinone rings, Bis-CoQ_0_ 3 > Bis-CoQ_0_ 2 > Bis-CoQ_0_ 1, confirmed by single crystal structures shown in Fig. 1^22–24^. Therefore, the electronic communications become feeble even nonexistent along with the increasing distance between the two quinonyl groups.

### Study of the comproportionation constant

The splitting degrees of the first two electron redox peaks of Bis-CoQ_0_ 1 and Bis-CoQ_0_ 2 also provide a direct approach for describing the thermodynamic stability of the monoradical (CoQ_0_-CoQ_0_^.−^), which is imparted by the electronic communications between two quinonyl groups during the redox processes and is related to the comproportionation constant Kc, Kc = exp[(E_1_^o^-E_2_^o^)n1n2F/RT] (In our case, n1 = n2 = 1, Kc = exp (ΔE^o^ / 25.69) at 298 K, with ΔE^o^ given in mV)^23^,^25^,^26^. From the separation of experimental and simulated reduction peaks (Fig. 2B), the comproportionation constants (Kc) of 2 × 10^3^ for CoQ_0_-CoQ_0_^.−^ 1 and 10 for CoQ_0_-CoQ_0_^.−^ 2 are calculated. The larger comproportionation constant indicates CoQ_0_-CoQ_0_^.−^ 1 is more stable than CoQ_0_-CoQ_0_^.−^ 2, in turn demonstrating the electronic communication is stronger in CoQ_0_-CoQ_0_^.−^ 1 than that in CoQ_0_-CoQ_0_^.−^ 2^26^,^27^.

### Density Functional Theory study of the redox processes

**Figure 3:**
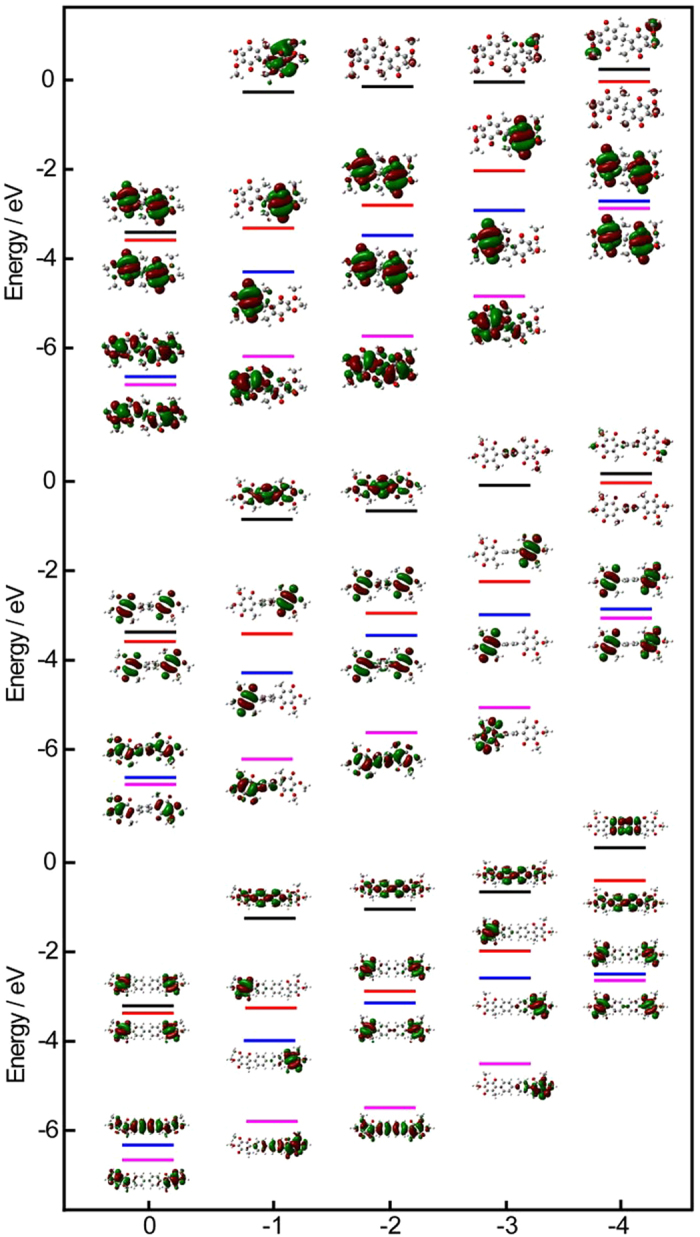
The energies of the frontier molecular orbitals (LUMO-1, black lines; LUMO, red lines; HOMO, blue lines; HOMO-1, pink lines) of three Bis-CoQ_0_s and their corresponding electronic density contours for neutral and different charge species in four electrons transfer processes.

Despite the different degrees of electronic communications in the redox processes of three Bis-CoQ_0_s can be explained by the increasing distance between the two peripheral ubiquinone groups, in order to better understand the effect of the structures on the electronic communications, we also carried out the Density Functional Theory (DFT) calculations for the four electrons transfer processes of Bis-CoQ_0_s at the b3lyp/6-311++g (d, p) level of theory and the integral equation formalism version of polarizable continuum model (IEF-PCM) was used for describing the solvent and the interaction between solvents and solutes, which were confirmed to accurately predict the reduction potentials of quinone related compounds^28^,^29^,^30^,^31^,^32^,^33^. The calculated energies and corresponding electronic density contours were shown in Table 2 and Fig. 3. As can be seen from Fig. 3, the electronic density contours of HOMO and HOMO-1 for three Bis-CoQ_0_s are almost localized on the same moieties in the four electrons transfer processes. However, the locations of electronic density contours of LUMO and LUMO+1, especially the LUMO+1, are very different, which may be contributed to the significant different in the electronic communications for three Bis-CoQ_0_s. The differences in these electronic density contours could be further confirmed by the energies of the HOMO, LUMO and the molecular orbital elements V_L_ (half of the energy splitting of the LUMO and LUMO+1 molecular orbitals) for electron transfer and V_H_ (half of the energy splitting of the LUMO and LUMO+1 molecular orbitals) for hole transfer as listed in Table 2. As shown in Table 2, the energies of LUMO and HOMO of three Bis-CoQ_0_s are almost same and decrease with the four electrons redox processes. Only the molecular orbital elements V_L_ for electron transfer has a notably and regular change in the four-electron transfer processes of three Bis-CoQ_0_s. The values of V_L_ are small in the neutral states of three Bis-CoQ_0_s and reach the maximum when the Bis-CoQ_0_s got one electron to form CoQ_0_-CoQ_0_^.−^s, then V_L_ gradually decreases accompany with the further reduction processes. The changes of V_L_ are consistent with the observed electrochemical behaviors of CoQ_0_-CoQ_0_s, especially the splitting of redox peaks for the first two electrons transfer processes. The values of V_L_ for three CoQ_0_-CoQ_0_^.−^s are 1.514 eV, 1.278 eV and 1.003 eV, which is agreement with the splitting degrees of the first and second redox peaks of Bis-CoQ_0_s as shown in Fig. 2. CoQ_0_-CoQ_0_^.−^ 1 has the highest V_L_ (1.514 eV), therefore the electronic communication is strongest in the two quinonyl groups and the peaks separation is large between CoQ_0_-CoQ_0_^.−^ and CoQ_0_^.−^-CoQ_0_^.−^. CoQ_0_-CoQ_0_^.−^ 2 has the smaller V_L_ (1.278 eV) than CoQ_0_-CoQ_0_^.−^ 1, therefore the electronic communication is weak and the peaks separation is small between CoQ_0_-CoQ_0_^.−^ and CoQ_0_^.−^-CoQ_0_^.−^ to form a merging peak. While the V_L_ of CoQ_0_-CoQ_0_^.−^ 3 (1.003 eV) is the smallest in three CoQ_0_-CoQ_0_^.−^s, there is no electron communication in the two quinonyl groups and Bis- CoQ_0_ 3 displays the similar electronchemical properties like CoQ_0_. These results indicate V_L_ has a directly relationship with the electronic communications between two quinonyl groups during the redox processes, which results in the different splitting degrees of redox peaks. In addition, according to the electrochemical data, the intramolecular electronic communication is stronger in the former two-electron transfer processes than the latter two-electron transfer processes due to the significant splitting of the first two electron reduction processes. Namely, the intramolecular electronic communication is strong after the generation of monoradicals (CoQ_0_-CoQ_0_^.−^) resulting in more negative reduction potential from CoQ_0_-CoQ_0_^.−^ to CoQ_0_^.−^-CoQ_0_^.−^ and becomes week during the reduction of diamagnetic dianions (CoQ_0_^.−^-CoQ_0_^.−^) to form tetraanions (CoQ_0_^2−^-CoQ_0_^2−^). The changed electronic communications are matched with the gradually decreasing V_L_ during the further redox processes. Together these results indicate the structures lead to the different in the molecular orbital elements V_L_ being responsible for three different degrees of intramolecular electronic communications in the redox processes of three Bis-CoQ_0_s. The intensity of intramolecular electronic communications in the redox processes are different^24^ and also could be measured by the V_L_ of the reduced intermediate species of Bis-CoQ_0_s. From these results, it is clear that V_L_ could be used for describing the electron communications between two peripheral groups during their redox processes.

### Solvents Effect on the Intramolecular Electronic Communications of Bis-CoQ_0_s

In order to further understand and tune the intramolecular electronic communications of three Bis-CoQ_0_s, we measured their electrochemical behaviors in five aprotic organic solvents including tetrahydrofuran (THF), dichloromethane (DCM), acetonitrile (AN), dimethylformamide (DMF) and dimethyl sulfoxide (DMSO) using CV and DPV techniques. The important solvent parameters are listed in Table S1.

**Figure 4:**
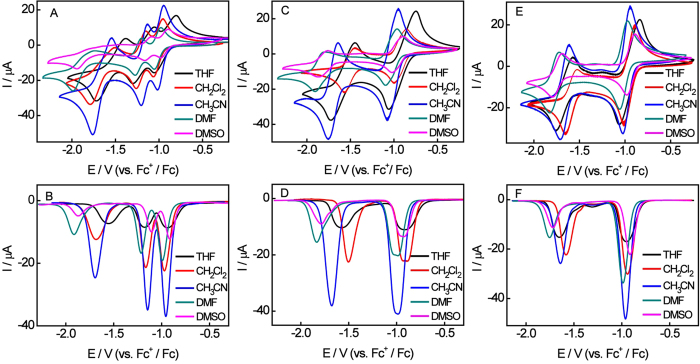
CV and DPV curves of 1.0 mM Bis-CoQ_0_ 1 (A and B), Bis-CoQ_0_ 2 (C and D) and Bis-CoQ_0_ 3 (E and F) obtained at a glassy carbon electrode in distilled THF (black line), CH_2_Cl_2_ (red line), CH_3_CN (blue line), DMF (orange line) and DMSO (pink line) containing 0.1 M TBAP at scan rates 0.010 Vs^−1^.

As shown in Fig. 4, for Bis-CoQ_0_ 1, solvent effects on the third step reduction process are more significant than the first and second reduction steps and the maximal potential shift for the three reduction peaks are 50 mV, 106 mV and 362 mV in the five solvents, respectively (Table S2). The similar phenomenon appears in Bis-CoQ_0_ 2 and Bis-CoQ_0_ 3, where solvent effects on the second reduction peak are more appreciable than the first reduction step and the maximal potential shift for two reduction peaks are 92 mV, 325 mV and 81 mV, 181 mV, respectively (Table S3 and S4). These results were confirmed by previous work that the solvent effect is minor for monoradicals so long as a tetra-alkylammonium salt is used as the supporting electrolyte and the effects is more significant on latter electron transfer processes that mainly rely on the nature of solvents (i.e. polarity, donor number)^34^. These electrochemical results also reveal that the solvent effect on the intramolecular electronic communication is week, especially the splitting of the former two-electron transfer. Even so, the solvents effect on the electronic communications of Bis-CoQ_0_ 2 is more interesting. From the DPV curves of Fig. 4D, the splitting of first reduction peak of Bis-CoQ_0_ 2 shows the different divisive degree in various solvents. This reduction peak splits into two peaks in CH_2_Cl_2_ and DMF indicating that the monoradical anion of Bis-CoQ_0_ 2 is greater stable in CH_2_Cl_2_ and DMF and has longer lift time^35^.

For the DPV studies of three Bis-CoQ_0_s in Fig. 4B,D,F, the reduction peak current is different in various aprotic solvents and the relative order is as follow CH_3_CN > CH_2_Cl_2_ > DMF > DMSO >THF, for the difference in diffusion coefficients of Bis-CoQ_0_s in the different solvents due to changing viscosities. As the solvent viscosity increased, the reduction peak currents decrease except for THF.

The first oxidation peak of Bis-CoQ_0_ 1 is symmetrical with the corresponding reduction peak in THF, CH_2_Cl_2_ and CH_3_CN, but it becomes asymmetric in DMF and almost disappears in DMSO. The result proclaims the latter two-electron transfer process of Bis-CoQ_0_ 1 appears serious chemically irreversible in DMF and DMSO than in other solvents. These results indicate chemical reactions between solvents and electrode products are more favorable in DMSO and DMF because of the large polarity and DN for DMF and DMSO^21^. In addition, the carbon atom in DMF and DMSO is electropositive, while the electrode product is electronegative, therefore more favorable reaction occurs between the solvent and electrode product. The same phenomenon occurs for Bis-CoQ_0_ 2 and Bis-CoQ_0_ 3, but interestingly, the oxidation peak did not disappear in DMSO. This result demonstrates the chemical reactions are influenced not only by the solvents but also by the electrode products^34^,^36^.

### DFT Study of Solvents Effect

To investigate the correlation and otherness between the experimental and the theoretically calculated electrode potentials in the five solvents, we carried out the DFT calculations for the four electrons transfer processes of Bis-CoQ_0_ 1 and Bis-CoQ_0_ 2 at the b3lyp/6-311++g (d, p) lever of theory with the IEF-PCM for describing the solvents. The Gibbs free energies were calculated using DFT, and then the reduction potentials were obtained based on the thermodynamic cycles^37^,^38^. Tables S5 and Table S7 display the calculated solvent phase Gibbs free energies for the species of Bis-CoQ_0_ 1 and Bis-CoQ_0_ 2 during the four-electron reduction processes. Table S6 and Table S8 list the calculated reduction potentials (Ecalc.) and experimental electrode potentials (Eexp.) of the four electrons reduction processes in five solvents, respectively.

**Figure 5:**
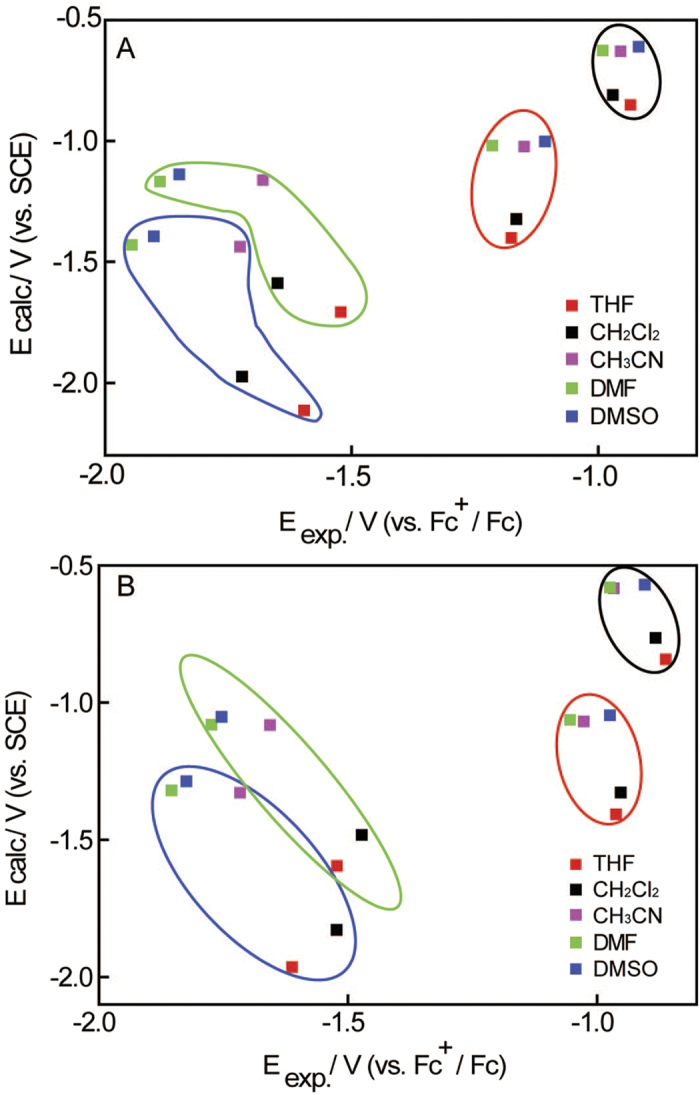
The scatter diagram of experimental vs. calculated reduction potentials for four electrons transfer processes of Bis-CoQ_0_ 1 (**A**) and Bis-CoQ_0_ 2 (**B**) in five aprotic solvents.

Figure 5 shows the correction and otherness of experimental and calculated electrode reduction potentials for the four electrons transfer processes of Bis-CoQ_0_ 1 and Bis-CoQ_0_ 2 in five solvents, respectively. It can be seen from Fig. 5, there are not significant relationships between the experimental and calculated reduction potentials owing to the fact that the conditions for experimental and calculated processes are not exactly same. Some complicated chemical reactions occurred in electrons transfer processes and we have not method to simulate these reactions during the calculations^21^. However, several trends are very clear in the Fig. 5. Firstly, the calculated results also indicated that the solvents effect on the latter two electrons transfer is more significant than the former two electrons transfer. Secondly, the calculated reduction potentials are almost same in CH_3_CN, DMF and DMSO but very different in THF and CH_2_Cl_2_, which can explained by the similar solvent parameters for CH_3_CN, DMF and DMSO, such as dielectric constant and polarity. Thirdly, the calculated reduction potentials in CH_3_CN, DMF and DMSO are similar for the first two electron transfer of Bis-CoQ_0_ 1 and Bis-CoQ_0_ 2, which also reveals that the solvents effect on the intramolecular electronic communication is feeble. These electrochemical results and DFT calculations indicate the solvents affect on the intramolecular electronic communications of three Bis-CoQ_0_s is very week and mostly rely on the nature of the solvents.

## Conclusion

In conclusion, the present work shows three degrees of intramolecular electronic communications in ubiquinone-based biomolecular wires (Bis-CoQ_0_s) measured using the electrochemical methods. The results indicate the bridge linkers could fine-tune the electronic communications between two peripheral ubiquinone groups, which is attributed to the different bridges linkers leading the changes in the molecular orbital elements V_L_ for electron transfer. Solvents display limited capability to tune intramolecular electronic communications of Bis-CoQ_0_s. The electrochemical and DFT studies indicate the solvents effect on the latter two electron transfer processes is more appreciable than the former two electron processes, which relies on the nature of these solvents and electrode products. In addition, the intensity of intramolecular electronic communications would change accompanying with reduction processes of Bis-CoQ_0_s, which also can be estimated using the V_L_ of reduced intermediate species. In future work, the long-distance electronic communications and great potential for implementation in artificial photosynthetic systems and electronic devices will be explored in quinone-based biomolecular wires.

## Methods

### Reagents and apparatus

HPLC-grade acetonitrile (CH_3_CN) and tetrabutylammonium perchlorate (TBAP, 98%) were purchased from Sigma-Aldrich. N_2_ (99.998%, prepurified) was gained from Cryogenic Gases. All the chemical reagents for synthesis and analysis were analytical grade, obtained from commercial suppliers, and used without further purification unless specified. All electrodes for electrochemical experiments were purchased from Shanghai Chenhua Co., Ltd., China. ^1^H NMR and ^13^C NMR were acquired in CDCl_3_ on BRUKER AVANCE 500 spectrometer using TMS as an internal standard. Mass spectrum was obtained on HP 5989 mass spectrometer.

### Synthesis

The synthetic routes of bis(2,3-dimethoxy-5-methyl-l,4-benzoquinone)methane (Bis-CoQ_0_ 1) had been reported in the literature^21^.

### Synthesis of Bis(2,3-dimethoxy-5-methyl-l,4-benzoquinone)phenylene (Bis-CoQ_0_ 2)

6-Bormoubiquinone (0.70 g, 2.68 mmol)^39^, 1,4-phenyldiboronic acid (0.21 g, 1.34 mmol) were dissolved in dioxane (40 mL), then K_2_CO_3_ (1.44 g, 10.43 mmol) was added and the mixture was degassed. Pd(PPh_3_)_4_ (0.09 g, 7.79 × 10-2 mmol) were added and the reaction mixture was stirred and refluxed overnight at 102 °C under nitrogen atmosphere. The mixture was cooled to room temperature and diluted with water (10 mL), then extracted with CH_2_Cl_2_ (20 mL × 3). The combined organic phases were washed brine and dried over anhydrous Na_2_SO_4_. After evaporation of the solvent, the oily residue was purified by column chromatography to afford Bis-CoQ_0_ 2 as a red solid^40^,^41^. The structure of Bis-CoQ_0_ 2 was confirmed by ^1^H and ^13^C NMR spectroscopy and MS. ^1^H NMR (500.0MHz, CDCl_3_, 298 K): 7.23 (s, 4H, due to 4×Ar-H), 4.09 (s, 6H, due to 2×–OCH_3_), 4.03 (s, 6H, 2×-OCH_3_), 2.00 (s, 6H, 2×-CH_3_) ppm; ^13^C NMR (125.7 MHz): 184.65 (C = O), 183.46 (C = O), 144.96 (C, quinonyl ring), 144.18 (C, quinonyl ring), 141.18 (C, quinonyl ring), 140.29 (C, quinonyl ring), 132.85 (C, benzene ring), 129.47 (C, benzene ring), 61.39 (4×-OCH_3_ ), 14.01 (2×-CH_3_) ppm; Mass spectrum: calculated for C24H22O8 438.1, found 438.1.

### Synthesis of Bis(2,3-dimethoxy-5-methyl-l,4-benzoquinone)biphenylene (Bis-CQ_0_ 3)

Bis-CQ_0_3 was synthesized as the same way of Bis-CQ_0_ 2. The structure of Bis-CoQ_0_ 3 was confirmed by ^1^H and ^13^C NMR spectroscopy and MS. 1H NMR (500.0MHz, CDCl_3_, 298 K): 7.68 (s, 4H, due to 4×Ar-H), 7.24 (s, 4H, due to 4×Ar-H), 4.08 (s, 6H, due to 2×–OCH_3_), 4.03 (s, 6H, 2×-OCH_3_), 2.01 (s, 6H, 2×-CH_3_) ppm; ^13^C NMR (125.7 MHz): 184.37 (C = O), 183.71 (C = O), 144.96 (C, quinonyl ring), 144.12 (C, quinonyl ring), 141.42 (C, quinonyl ring), 140.79 (C, quinonyl ring), 140.03 (C, quinonyl ring), 131.82 (C, quinonyl ring), 130.25 (C, benzene ring), 126.88 (C, benzene ring), 61.39 (4×-OCH_3_), 13.96 (2×-CH_3_) ppm; Mass spectrum: calculated for C30H26O8 514.2(100.0%), found 514.2.

### Preparation of single crystals

Crystals of three Bis-CoQ_0_s were grown via slow diffusion of a dichloromethane solution with petroleum ether at 25 °C over a few days.

### Electrochemical measurements

A three-electrode cell was used for electrochemical measurements; glassy carbon (3 mm diameter) electrode, platinum wire and Ag/AgCl wire electrode were used as the working, counter and quasi-reference electrodes, respectively. CH_2_Cl_2_, CH_3_CN, DMF and DMSO were initially dried by distillation over CaH_2_, and THF was dried over Na, before the electrochemistry experiments. The measurements were carried out in five solvents containing 1.0 mM Bis-CoQ_0_s and 0.1 M TBAP. Accurate potentials were gained by using ferrocenium/ferrocene as an internal standard. During the measurement, a dry nitrogen purge maintained an oxygen and moisture free environment and the temperature were controlled in 25 °C by using circulating water bath. All the electrochemistry measurements were performed at CHI 660 electrochemical work station (Shanghai Chenhua Co., Ltd., China).

### Electrochemical Peak Fitting Method

Simulations were made out using the autonomous software, which can be used to digitally simulate the common peak fitting experiments. We used our previous reported method to obtain the simulated reduction potentials of CoQ_0_ and three Bis-CoQ_0_s^21^.

### Theoretical Computations

Density Functional Theory (DFT) was carried out using the Gaussian 09 software. The geometries of all species of Bis-CoQ_0_ 1, Bis-CoQ_0_ 2 and Bis-CoQ_0_ 3 in the redox processes were optimized at the b3lyp/6-311++g (d, p) level of theory, and the vibrational frequencies were also achieved at this level. To calculate the Gibbs free energy of every species in solvent at ambient temperature, the integral equation formalism version polarizable continuum model (IEF-PCM) implemented in Gaussian 09 codes was used for describing the solvent and the interaction between solvents and solutes. The integral equation formalism version of PCM (IEF-PCM) was used, which builds up the atomic radii by Universal Force Field (UFF) and cavity using a Scaled van der Waals Surface (VdW) (Alpha = 1.100) model. The temperature, 298.15 k, is same in the calculations and our electrochemical experiments. The rest of the computational parameters in the solvation models have been kept as default values. The reduction potentials were calculated based on the thermodynamic cycles.

Thermodynamic cycles of reduction processes for Bis-CoQ_0_^37^,^38^.
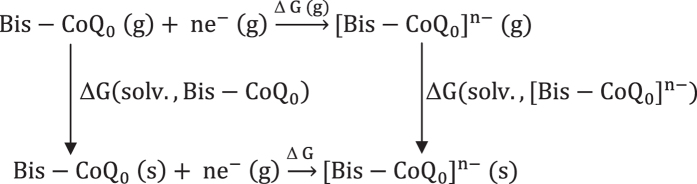


The reduction potentials of Bis-CoQ_0_ can be gained from the change in Gibbs free energy of the reduction processes, as shown in equation 1:
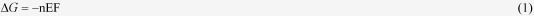
Where n is the number of electrons transferred and F is the Faraday constant.

As shown in the thermodynamic cycles, ΔG of the reduction processes can be calculated from below equation 2:
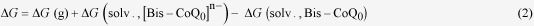
Where ΔG(g), calculated using equation 3, is the change of Gibbs free energy of reduction processes in the gas phase, and ΔG(solv., [Bis-CoQ_0_]^n-^) and ΔG(solv., [Bis-CoQ_0_]) are solvation energies of [Bis-CoQ_0_]^n-^ and Bis-CoQ_0_ in solvent, respectively. ΔG(solv., [Bis-CoQ_0_]^n-^) and ΔG(solv., [Bis-CoQ_0_]) can be obtained via equations 4 and 5.
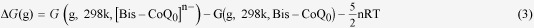

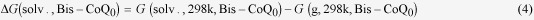

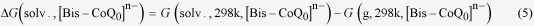
Where G(solv., [Bis-CoQ_0_]^n-^) and ΔG(g., [Bis-CoQ_0_]^n-^) are the Gibbs free energies of [Bis-CoQ_0_]^n-^ in gas and solvent phase, and 5/2 nRT is the thermal energy of free electrons.

Then the ΔG can be simplified as below equation 6:
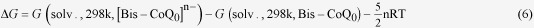


Hence, the calculated reduction potential relative to standard carbon electrode was obtained by equation 7:
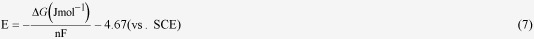


Molecular orbital elements V_L_ for electron transport (hole transport V_H_) reported in Table 1 was calculated as half the energy difference between the LUMO and LUMO+1 (HOMO and HOMO-1).

## Additional Information

**How to cite this article**: Liu, X.-Y. *et al*. Bridge- and Solvent-Mediated Intramolecular Electronic Communications in Ubiquinone-Based Biomolecular Wires. *Sci. Rep.* **5**, 10352; doi: 10.1038/srep10352 (2015).

## Supplementary Material

Supporting Information Supplementary Figures 1 and Supplementary Tables 1-6

## Figures and Tables

**Table 1:** The area and the half-width (W1/2) of each reduction peak of different compounds.

	**CoQ**_**0**_		**Bis-CoQ**_**0**_ **1**			**Bis-CoQ**_**0**_ **2**		**Bis-CoQ**_**0**_ **3**	
	**I**	**II**	**I**	**II**	**III**	**I**	**II**	**I**	**II**
A	5.05	4.60	4.27	3.67	3.80	7.04	5.80	5.67	3.43
W1/2 (mv)	94	113	94	113	140	170	140	94	113

**Table 2:** The energies (eV) of the HOMO, LUMO and the molecular orbital elements V_L_ for electron transfer and V_H_ for hole transfer of neutral and different charge species in four electrons transfer processes of three Bis-CoQ_0_s.

	**Bis-CoQ**_**0**_ **1**				**Bis-CoQ**_**0**_ **2**				**Bis-CoQ**_**0**_ **3**			
	**V**_**L**_	**E**_**LUMO**_	**E**_**HOMO**_	**V**_**H**_	**V**_**L**_	**V**_**LUMO**_	**E**_**HOMO**_	**V**_**H**_	**V**_**L**_	**V**_**LUMO**_	**E**_**HOMO**_	**V**_**H**_
0	0.018	−3.536	−6.724	0.057	0.021	−3.526	−6.709	0.048	0.011	−3.320	−6.355	0.130
−1	1.514	−3.320	−4.331	0.909	1.278	−3.430	−4.321	0.956	1.003	−3.266	−4.018	0.879
−2	1.335	−2.810	−3.500	1.104	1.150	−2.985	−3.482	1.068	0.912	−2.878	−3.175	1.137
−3	0.992	−2.034	−2.906	0.946	1.066	−2.246	−3.020	0.993	0.649	−1.982	−2.626	0.947
−4	0.098	0.041	−2.738	0.019	0.074	−0.033	−2.935	0.014	0.377	−0.415	−2.527	0.040
