# Supplementary material for: Bridge- and Solvent-Mediated Intramolecular Electronic Communications in Ubiquinone-Based Biomolecular Wires
Source: Sci Rep. 2015 May 21;5:10352. doi: 10.1038/srep10352 (PMC4440530; doi:10.1038/srep10352)
Supplement: Supporting Information — Supplementary Figures 1 and Supplementary Tables 1-6 [file srep10352-s1.pdf]

Supporting Information for

Bridge- and Solvent-Mediated Intramolecular Electronic Communication in Ubiquinone-Based Biomolecular Wires

Xiao-Yuan Liu[1], Wei Ma[1], Hao Zhou[1], Xiao-Ming Cao[1] & Yi-Tao Long[1]

[1] Key Laboratory for Advanced Materials & Department of Chemistry, East China University of Science and Technology, Shanghai 200237, P. R. China.

Table of Contents

NMR and MS Spectra **S3**

Electrochemistry Studies **S6**

DFT Calculations **S8**

References **S11**

Single Crystal Data **S12**

NMR and MS Spectra

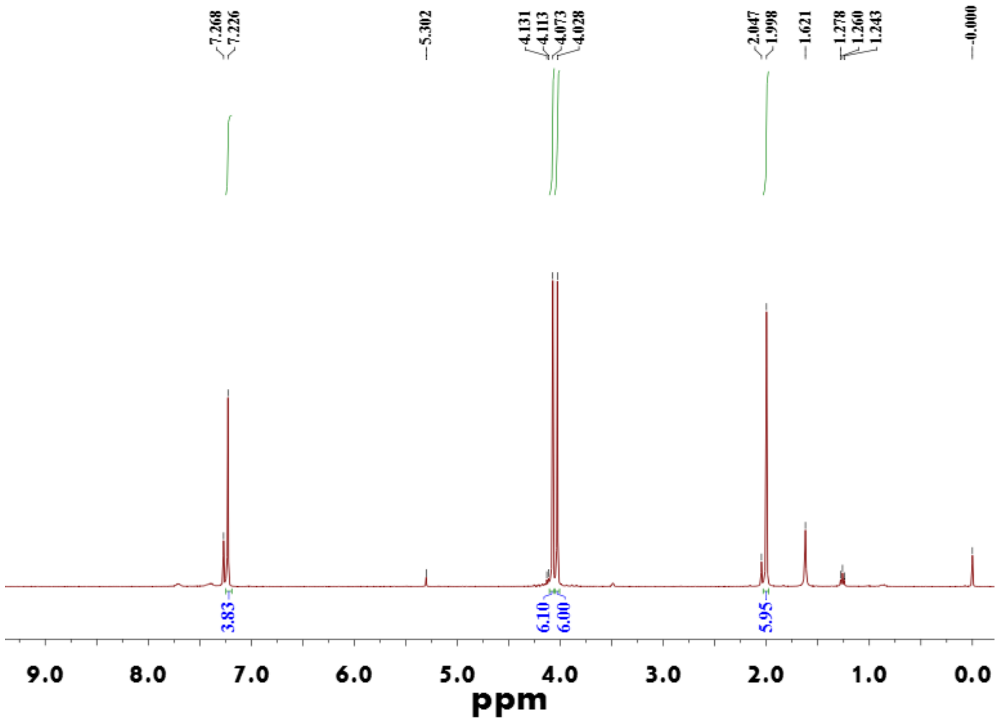

Figure S1. ${}^{1}H$ NMR spectrum of Bis-CoQ$_0$ **2** in $CDCl_3$

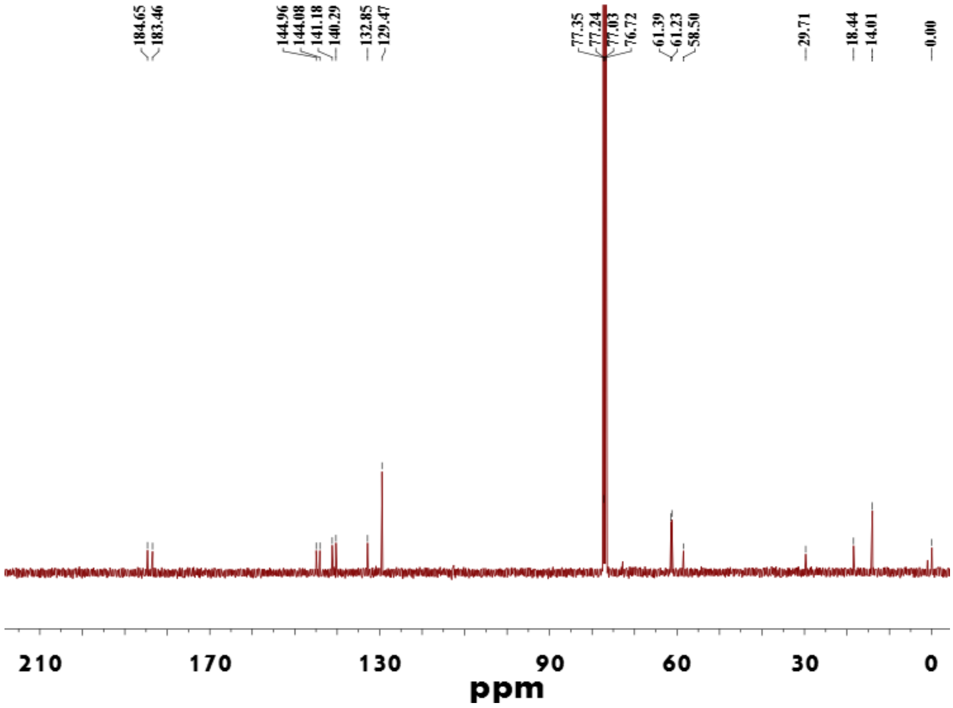

Figure S2. ${}^{13}C$ NMR spectrum of Bis-CoQ$_0$ **2** in $CDCl_3$

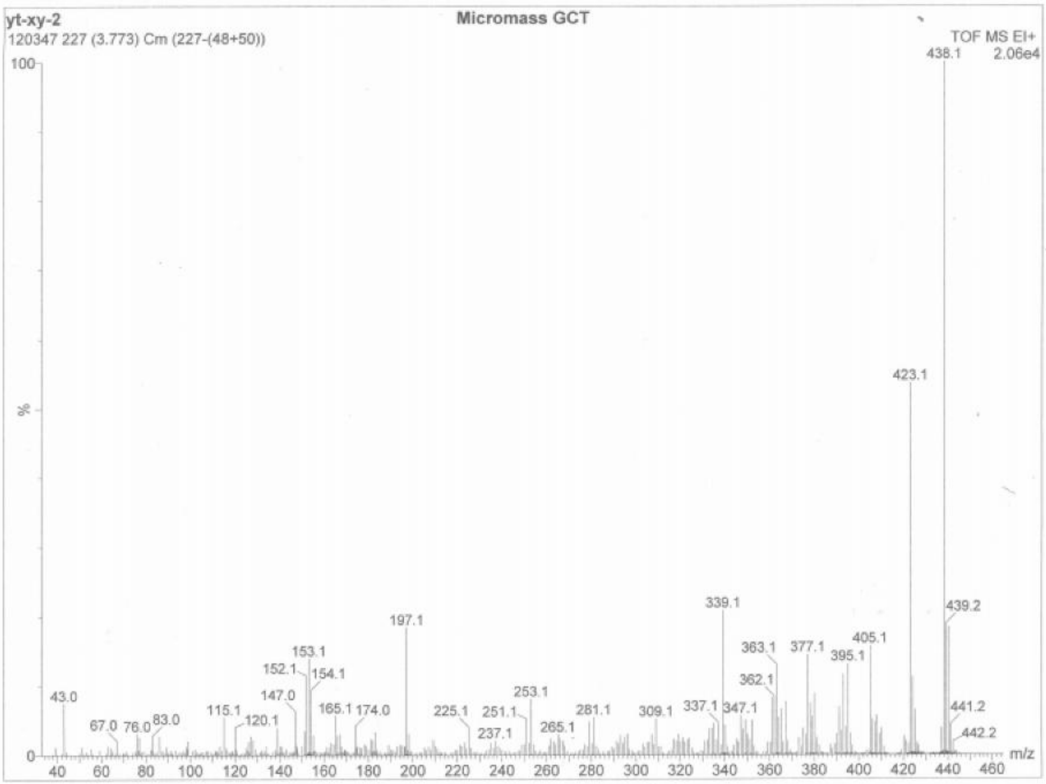

Figure S3. Mass spectrum of Bis-CoQ$_0$ **2** in $CDCl_3$

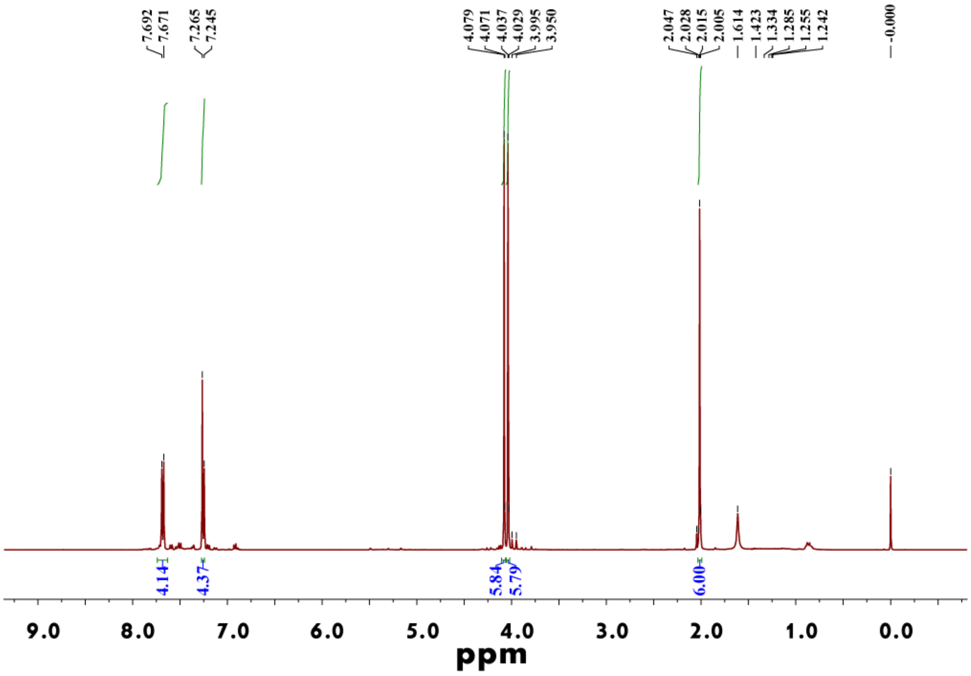

Figure S4. ^{1}H NMR spectrum of Bis-CoQ$_0$ **3** in $CDCl_3$

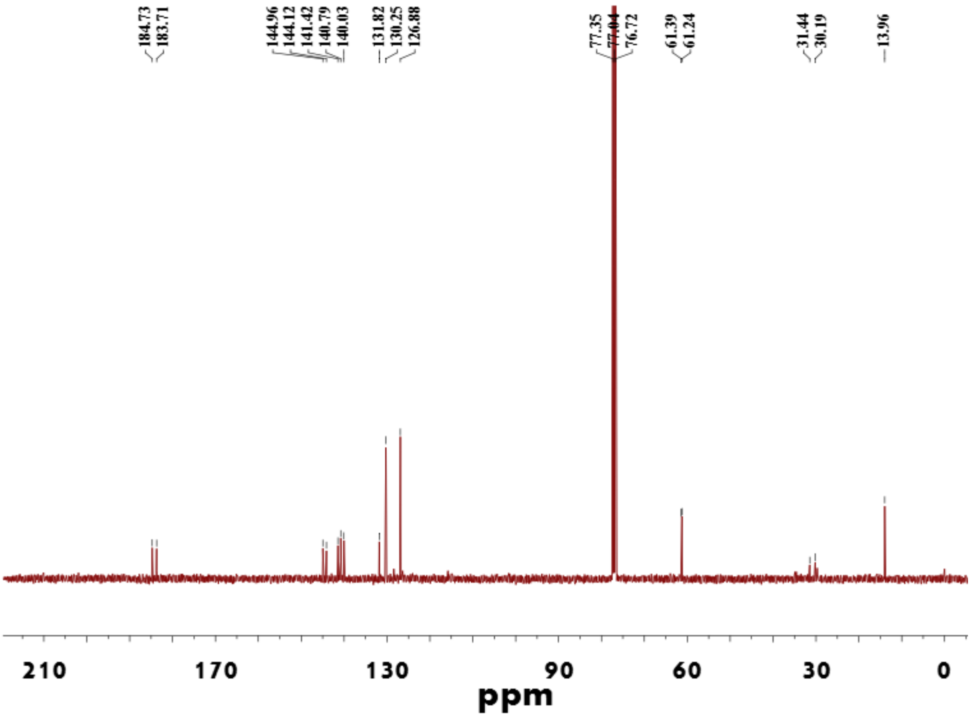

Figure S5. ^{13}C NMR spectrum of Bis-CoQ_0 **3** in $CDCl_3$

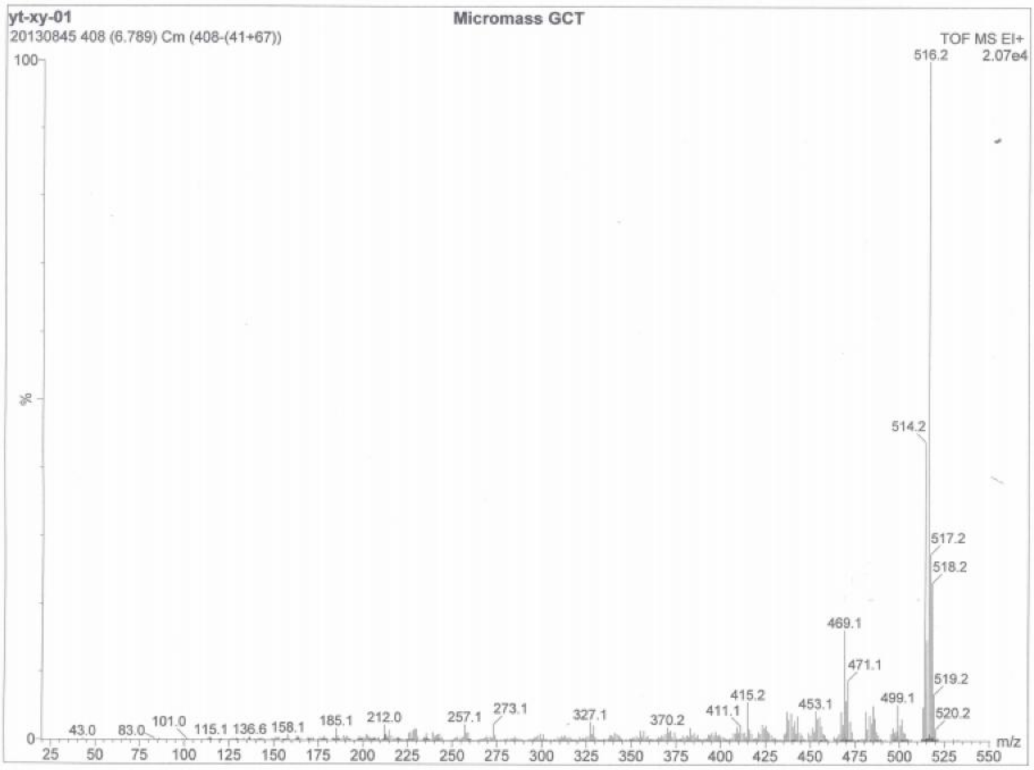

Figure S6. Mass spectrum of Bis-CoQ_0 **3** in $CDCl_3$

Electrochemistry Studies

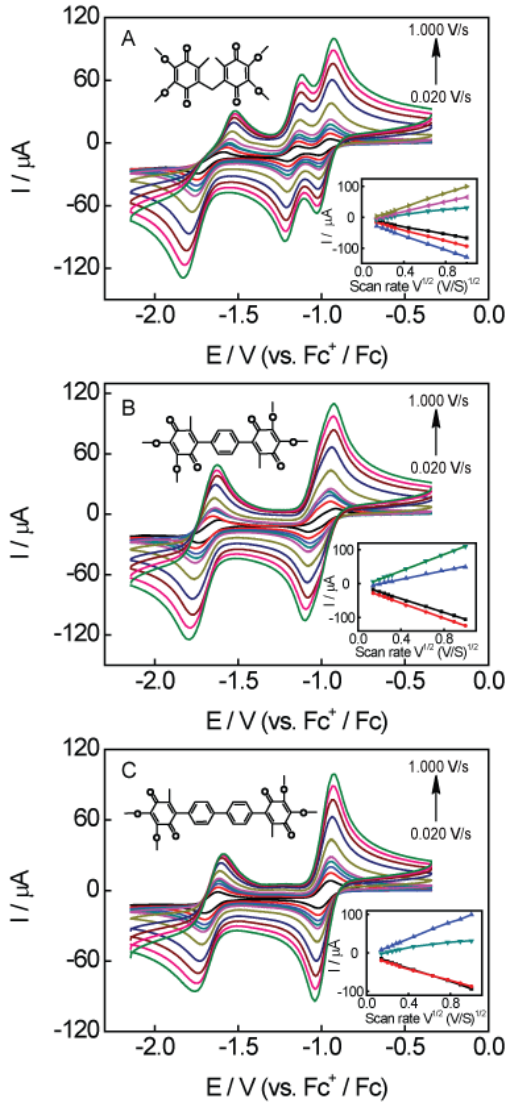

Figure S7. CV curves of 1.0 mM Bis-CoQ_0 **1** (A), Bis-CoQ_0 **2** (B) and Bis-CoQ_0 **3** (C) obtained at GC electrode in distilled CH_3CN containing 0.1 M TBAP at different scan rates of 0.020, 0.040, 0.060, 0.080, 0.100, 0.200, 0.400, 0.600, 0.800 and 1.000 Vs^{-1}. The inset figure shows the linear increasing of the redox peak current versus the square root of the scan rate.

The important solvent parameters, namely, dielectric constant (ε), donor number (DN), acceptor number (AN), polarity and viscosity, are listed in Table 1 for the five aprotic organic solvents chosen for this study[1, 2].

Table S1. Relevant Solvent Parameter

Solvent	ε^T	DN	AN	polarity	viscosity(mPa·s)
THF	7.52[22]	20.0	8.0	4.2	0.462
DCM	8.93[25]	(0)	20.4	3.4	0.411
AN	36.6[20]	14.1	18.9	6.2	0.341
DMF	38.2[20]	26.6	16.0	6.4	0.802
DMSO	47.2[20]	29.8	19.3	7.2	1.987

All the standard potential values of Bis-CoQ_0 1 and 2 are listed in Table S2 and S3, where the standard potential separation $\Delta E_{1/2}$ between adjacent two peaks is also listed.

Table S2. Redox Potentials versus Fc^+/ Fc for Bis-CoQ_0 **1** in five aprotic solvents.

Solvent	$E_{1/2}$(1,V)	$E_{1/2}$(2,V)	$\Delta E_{1/2}^a$(mV)	$E_{1/2}$(3/4,V)	$\Delta E_{1/2}^b$(mV)
Tetrahydrofuran	-0.929	-1.176	247	-1.551	375
Dichloromethane	-1.010	-1.207	197	-1.630	423
Acetonitrile	-0.980	-1.167	187	-1.653	486
DMF	-1.015	-1.236	221	-1.864	628
DMSO	-0.937	-1.123	186	-1.689	556

a : $\Delta E_{1/2}^a = E_{1/2}(1,V) - E_{1/2}(2,V)$;

b : $\Delta E_{1/2}^b = E_{1/2}(2,V) - E_{1/2}(3/4,V)$.

Table S3. Redox Potentials versus Fc^+/ Fc for Bis-CoQ_0 **2** in five aprotic solvents.

Solvent	$E_{1/2}$(1/2,V)	$E_{1/2}$(3/4,V)	$\Delta E_{1/2}^c$(mV)
Tetrahydrofuran	-0.903	-1.587	684
Dichloromethane	-0.925	-1.505	580
Acetonitrile	-1.012	-1.697	685
DMF	-1.028	-1.799	771
DMSO	-0.957	-1.814	857

c : $\Delta E_{1/2}^c = E_{1/2}(1/2,V) - E_{1/2}(3/4,V)$.

Table S4. Redox Potentials versus Fc^+/ Fc for Bis-CoQ_0 **3** in five aprotic solvents.

Solvent	$E_{1/2}$(1/2,V)	$E_{1/2}$(3/4,V)	$\Delta E_{1/2}^c$(mV)
Tetrahydrofuran	-0.949	-1.663	714

Dichloromethane	-0.950	-1.592	642
Acetonitrile	-0.984	-1.661	677
DMF	-1.016	-1.773	789
DMSO	-0.935	-1.741	806

d : $\Delta E_{1/2}{}^{d} = E_{1/2}(1/2,V) - E_{1/2}(3/4,V)$.

Electrochemical Peak Fitting Method

Simulations were made out using the autonomous software, which can be used to digitally simulate the common peak fitting experiments. We used our previous reported method[3] to obtain all the simulated reduction potentials of Bis-CoQ$_0$ 1 and Bis-CoQ$_0$ 2 (Table S6 and Table S8).

Theoretical Computations

Density functional theory (DFT) was carried out using the Gaussian 09 software. Geometries of all species of **Bis-CoQ$_0$ 1** and **Bis-CoQ$_0$ 2** in the redox processes were optimized at the b3lyp/6-311++g (d, p) lever of theory, and the nature of each stationary point was established by b3lyp/6-311++g (d, p) frequency calculations. To calculate the Gibbs free energy of every species in solvent, polarizable continuum model (PCM) was used to describe the solvent and the interaction between solvents and solutes.

The reduction potentials of Bis-CoQ$_0$ 1 and Bis-CoQ$_0$ 2 were calculated using the thermodynamic cycles shown in Scheme 1[4].

Scheme 1. Thermodynamic cycles of reduction processes for Bis-CoQ$_0$.

$$\begin{array}{ccc}
\mathrm{Bis-CoQ_0\ (g) + ne^-\ (g)} & \xrightarrow{\Delta\,G\,(g)} & \mathrm{[Bis-CoQ_0]^{n-}\ (g)} \\
\Big\downarrow \Delta G(\mathrm{solv., Bis-CoQ_0}) & & \Big\downarrow \Delta G(\mathrm{solv., [Bis-CoQ_0]^{n-}}) \\
\mathrm{Bis-CoQ_0\ (s) + ne^-\ (g)} & \xrightarrow{\Delta\,G\,(g)} & \mathrm{[Bis-CoQ_0]^{n-}\ (s)}
\end{array}$$

The reduction potentials of Bis-CoQ$_0$ can be gained from the change in Gibbs free energy of the

reduction processes, as shown in equation 1:

$$\Delta G = -nEF \qquad (1)$$

Where n is the number of electrons transferred and F is the Faraday constant.

As shown in Scheme 2, ΔG of the reduction processes can be calculated from below equation 2:

$$\triangle G = \triangle G\,(g) + \triangle G\,(solv., [Bis\text{–}CoQ_0]^{n-}) - \triangle G\,(solv., Bis\text{–}CoQ_0) \qquad (2)$$

Where ΔG(g), calculated using equation 3, is the change of Gibbs free energy of reduction processes in the gas phase, and ΔG(solv., $[Bis\text{-}CoQ_0]^{n-}$) and ΔG(solv., [Bis-CoQ_0]) are solvation energies of $[Bis\text{-}CoQ_0]^{n-}$ and Bis-CoQ_0 in solvent, respectively. ΔG(solv., $[Bis\text{-}CoQ_0]^{n-}$) and ΔG(solv., [Bis-CoQ_0]) can be obtained via equations 4 and 5.

$$\triangle G(g) = G\,(g, 298k, [Bis\text{–}CoQ_0]^{n-}) - G\,(g, 298k, Bis\text{–}CoQ_0) - \frac{5}{2}nRT \qquad (3)$$

$$\triangle G(solv., Bis\text{–}CoQ_0) = G\,(solv., 298k, Bis\text{–}CoQ_0) - G\,(g, 298k, Bis\text{–}CoQ_0) \qquad (4)$$

$$\triangle G(solv., [Bis\text{–}CoQ_0]^{n-}) = G\,(solv., 298k, [Bis\text{–}CoQ_0]^{n-}) - G\,(g, 298k, [Bis\text{–}CoQ_0]^{n-}) \qquad (5)$$

Where G(solv., $[Bis\text{-}CoQ_0]^{n-}$) and ΔG(g., $[Bis\text{-}CoQ_0]^{n-}$) are the Gibbs free energies of $[Bis\text{-}CoQ_0]^{n-}$ in gas and solvent phase, and 5/2 nRT is the thermal energy of free electrons.

Then the ΔG can be simplified as below equation 6:

$$\triangle G = G\,(solv., 298k, [Bis\text{–}CoQ_0]^{n-}) - G\,(solv., 298k, Bis\text{–}CoQ_0) - \frac{5}{2}nRT \quad (6)$$

Hence, the calculated reduction potential relative to standard carbon electrode was obtained by equation 7:

$$E = -\frac{\Delta G(Jmol^{-1})}{nF} - 4.67(vs. SCE) \qquad (7)$$

Table S5. Calculated Gibbs free energies[a] for four-electron transfer processes of Bis-CoQ_0 1 in five aprotic solvents at b3lyp/6-311++g (d, p) level of theory.

	THF	CH_2Cl_2	CH_3CN	DMF	DMSO
Bis-CoQ_0 1	-1337.831488	-1337.832355	-1337.835415	-1337.835462	-1337.835605
[Bis-CoQ_0 1]$^{-1}$	-1337.969462	-1337.971836	-1337.981538	-1337.981688	-1337.982392
[Bis-CoQ_0 1]$^{-2}$	-1338.067024	-1338.073618	-1338.098669	-1338.099009	-1338.100374
[Bis-CoQ_0 1]$^{-3}$	-1338.150945	-1338.164940	-1338.215103	-1338.214412	-1338.217829
[Bis-CoQ_0 1]$^{-4}$	-1338.197723	-1338.219048	-1338.301105	-1338.302200	-1338.307597

[a] In atomic units, Hartree (1 Hartree = 2625.49975 kJ mol^{-1}).

Table S6. Calculated and experimental (or simulated) reductions potentials for four-electron transfer processes of Bis-CoQ_0 1 in five aprotic solvents.

	THF		CH_2Cl_2		CH_3CN		DMF		DMSO	
	$E_{calc.}$(V)[b]	$E_{exp.}$ (V)[c]	$E_{calc.}$(V)[b]	$E_{exp.}$ (V)[c]	$E_{calc.}$(V)[b]	$E_{exp.}$ (V)[c]	$E_{calc.}$(V)[b]	$E_{exp.}$ (V)[c]	$E_{calc.}$(V)[b]	$E_{exp.}$ (V)[c]
[Bis-CoQ_0 1]$^{-1}$	-0.851	-0.934	-0.810	-0.970	-0.630	-0.954	-0.627	-0.991	-0.611	-0.916
[Bis-CoQ_0 1]$^{-2}$	-1.401	-1.176	-1.323	-1.166	-1.024	-1.150	-1.020	-1.215	-1.003	-1.106
[Bis-CoQ_0 1]$^{-3}$	-1.708	-1.522	-1.589	-1.650	-1.162	-1.680	-1.169	-1.889	-1.139	-1.848
[Bis-CoQ_0 1]$^{-4}$	-2.114	-1.596	-1.722	-1.722	-1.438	-1.726	-1.431	-1.946	-1.395	-1.900

[b] vs. SCE.

[c] vs. Fc^+ / Fc.

Table S7. Calculated Gibbs free energies[a] for four-electron transfer processes of Bis-CoQ_0 2 in five aprotic solvents at b3lyp/6-311++g (d, p) level of theory.

	THF	CH_2Cl_2	CH_3CN	DMF	DMSO
Bis-CoQ_0 2	-1529.571859	-1529.571886	-1529.575852	-1529.575921	-1529.576242
[Bis-CoQ_0 2]$^{-1}$	-1529.710151	-1529.713083	-1529.723818	-1529.723979	-1529.724665
[Bis-CoQ_0 2]$^{-2}$	-1529.806934	-1529.812834	-1529.836154	-1529.836496	-1529.838122
[Bis-CoQ_0 2]$^{-3}$	-1529.903782	-1529.916252	-1529.964838	-1529.964915	-1529.968271
[Bis-CoQ_0 2]$^{-4}$	-1529.960228	-1529.980148	-1530.058216	-1530.059309	-1530.064663

[a] In atomic units, Hartree (1 Hartree = 2625.49975 kJ mol^{-1}).

Table S8. Calculated and experimental (or simulated) reductions potentials for four-electron transfer processes of Bis-CoQ_0 2 in five aprotic solvents.

	THF		CH_2Cl_2		CH_3CN		DMF		DMSO	
	$E_{calc.}$(V)[b]	$E_{exp.}$ (V)[c]	$E_{calc.}$(V)[b]	$E_{exp.}$ (V)[c]	$E_{calc.}$(V)[b]	$E_{exp.}$ (V)[c]	$E_{calc.}$(V)[b]	$E_{exp.}$ (V)[c]	$E_{calc.}$(V)[b]	$E_{exp.}$ (V)[c]
[Bis-CoQ_0 2]$^{-1}$	-0.843	-0.862	-0.764	-0.878	-0.579	-0.967	-0.577	-0.968	-0.567	-0.900
[Bis-CoQ_0 2]$^{-2}$	-1.407	-0.962	-1.327	-0.948	-1.064	-1.027	-1.060	-1.048	-1.043	-0.970
[Bis-CoQ_0 2]$^{-3}$	-1.595	-1.522	-1.482	-1.468	-1.077	-1.657	-1.077	-1.768	-1.049	-1.750

$[Bis\text{-}CoQ_0\ 2]^{-4}$	-1.964	-1.612	-1.828	-1.518	-1.324	-1.717	-1.317	-1.848	-1.283	-1.820

[b] vs. SCE.

[c] vs. Fc^+ / Fc.

References

1. Barrière, F.; Geiger, W. E. *J. Am. Chem. Soc.* **2006**, *128*, 3980.
2. Kauffman, G. W.; Jurs, P. C. *J. Chem. Inf. Comput. Sci.* **2001**, *41*, 408.
3. Wang, X.; Ma, W.; Ying, Y.; Liang, J.; Long, Y.-T. *Chem. Asian J* **2011**, *6*, 1064.
4. Namazian, M.; Coote, M. L. *J. Phys. Chem. A* **2007**, *111*, 7227.

Single Crystal Data

Single Crystal Data of Bis-CoQ$_0$ 1.

Table 1. Crystal data and structure refinement for Bis-CoQ$_0$ 1.

Identification code	mo_dm13389_0m	
Empirical formula	C19 H20 O8	
Formula weight	376.35	
Temperature	140(2) K	
Wavelength	0.71073 Å	
Crystal system	Monoclinic	
Space group	C 2/c	
Unit cell dimensions	a = 27.874(10) Å	α= 90 °.
	b = 4.3595(14) Å	β= 118.507(5) °.
	c = 16.358(5) Å	γ = 90 °.
Volume	1746.7(10) Å^3	
Z	4	
Density (calculated)	1.431 Mg/m^3	
Absorption coefficient	0.112 mm^{-1}	
F(000)	792	
Crystal size	0.300 x 0.030 x 0.020 mm^3	
Theta range for data collection	1.663 to 30.386 °.	
Index ranges	-38<=h<=39, -6<=k<=6, -21<=l<=23	
Reflections collected	7962	
Independent reflections	2616 [R(int) = 0.0588]	
Completeness to theta = 25.242 °	99.2 %	
Absorption correction	Semi-empirical from equivalents	
Max. and min. transmission	0.7461 and 0.5431	
Refinement method	Full-matrix least-squares on F^2	
Data / restraints / parameters	2616 / 0 / 126	
Goodness-of-fit on F^2	0.943	
Final R indices [I>2sigma(I)]	R1 = 0.0601, wR2 = 0.1624	
R indices (all data)	R1 = 0.1245, wR2 = 0.1992	
Extinction coefficient	n/a	
Largest diff. peak and hole	0.295 and -0.216 e.Å^{-3}	

Table 2. Atomic coordinates (x 10^4) and equivalent isotropic displacement parameters (Å^{2}x 10^3) for Bis-CoQ$_0$ 1. U(eq) is defined as one third of the trace of the orthogonalized U^{ij} tensor.

	x	y	z	U(eq)

O(1)	2027(1)	251(4)	4252(1)	51(1)
O(2)	1341(1)	180(5)	4882(1)	57(1)
O(3)	1748(1)	4047(4)	2585(1)	51(1)
O(4)	793(1)	7040(4)	1922(1)	59(1)
C(1)	2441(1)	-48(6)	3962(2)	52(1)
C(2)	1575(1)	1917(5)	3761(1)	40(1)
C(3)	1422(1)	3598(5)	2990(1)	41(1)
C(4)	904(1)	5325(5)	2576(2)	42(1)
C(5)	523(1)	5039(5)	2982(2)	39(1)
C(9)	0	6841(7)	2500	48(1)
C(7)	1209(1)	1742(5)	4194(1)	40(1)
C(6)	682(1)	3460(5)	3773(1)	38(1)
C(8)	362(1)	3274(6)	4298(2)	46(1)
C(10)	1548(1)	2738(7)	1670(2)	54(1)

Table 3. Bond lengths [Å] and angles [°] for Bis-CoQ$_0$ 1.

O(1)-C(2)	1.338(3)
O(1)-C(1)	1.449(3)
O(2)-C(7)	1.212(2)
O(3)-C(3)	1.373(3)
O(3)-C(10)	1.441(3)
O(4)-C(4)	1.219(3)
C(1)-H(1A)	0.9800
C(1)-H(1B)	0.9800
C(1)-H(1C)	0.9800
C(2)-C(3)	1.340(3)
C(2)-C(7)	1.497(3)
C(3)-C(4)	1.476(4)
C(4)-C(5)	1.502(3)
C(5)-C(6)	1.341(3)
C(5)-C(9)	1.506(3)
C(9)-C(5)#1	1.506(3)
C(9)-H(9A)	0.9900
C(9)-H(9B)	0.9900
C(7)-C(6)	1.493(3)
C(6)-C(8)	1.504(3)
C(8)-H(8A)	0.9800
C(8)-H(8B)	0.9800

C(8)-H(8C)	0.9800
C(10)-H(10A)	0.9800
C(10)-H(10B)	0.9800
C(10)-H(10C)	0.9800
C(2)-O(1)-C(1)	121.43(19)
C(3)-O(3)-C(10)	115.33(18)
O(1)-C(1)-H(1A)	109.5
O(1)-C(1)-H(1B)	109.5
H(1A)-C(1)-H(1B)	109.5
O(1)-C(1)-H(1C)	109.5
H(1A)-C(1)-H(1C)	109.5
H(1B)-C(1)-H(1C)	109.5
O(1)-C(2)-C(3)	130.3(2)
O(1)-C(2)-C(7)	110.36(19)
C(3)-C(2)-C(7)	119.3(2)
C(2)-C(3)-O(3)	123.1(2)
C(2)-C(3)-C(4)	120.91(19)
O(3)-C(3)-C(4)	115.75(19)
O(4)-C(4)-C(3)	119.9(2)
O(4)-C(4)-C(5)	120.2(2)
C(3)-C(4)-C(5)	119.86(19)
C(6)-C(5)-C(4)	119.5(2)
C(6)-C(5)-C(9)	124.37(18)
C(4)-C(5)-C(9)	115.90(17)
C(5)-C(9)-C(5)#1	117.1(3)
C(5)-C(9)-H(9A)	108.0
C(5)#1-C(9)-H(9A)	108.0
C(5)-C(9)-H(9B)	108.0
C(5)#1-C(9)-H(9B)	108.0
H(9A)-C(9)-H(9B)	107.3
O(2)-C(7)-C(6)	120.37(19)
O(2)-C(7)-C(2)	119.6(2)
C(6)-C(7)-C(2)	120.00(19)
C(5)-C(6)-C(7)	120.09(19)
C(5)-C(6)-C(8)	124.4(2)
C(7)-C(6)-C(8)	115.48(19)
C(6)-C(8)-H(8A)	109.5
C(6)-C(8)-H(8B)	109.5

H(8A)-C(8)-H(8B)	109.5
C(6)-C(8)-H(8C)	109.5
H(8A)-C(8)-H(8C)	109.5
H(8B)-C(8)-H(8C)	109.5
O(3)-C(10)-H(10A)	109.5
O(3)-C(10)-H(10B)	109.5
H(10A)-C(10)-H(10B)	109.5
O(3)-C(10)-H(10C)	109.5
H(10A)-C(10)-H(10C)	109.5
H(10B)-C(10)-H(10C)	109.5

Symmetry transformations used to generate equivalent atoms:

#1 -x,y,-z+1/2

Table 4. Anisotropic displacement parameters (Å^{2}x 10^3) for Bis-CoQ$_0$ 1. The anisotropic displacement factor exponent takes the form: $-2\pi^2$[h^2 a*^{2}U^{11} + ... + 2 h k a* b* U^{12}]

	U^{11}	U^{22}	U^{33}	U^{23}	U^{13}	U^{12}
O(1)	43(1)	75(1)	40(1)	-2(1)	23(1)	-5(1)
O(2)	49(1)	89(1)	38(1)	14(1)	25(1)	1(1)
O(3)	52(1)	71(1)	41(1)	-14(1)	30(1)	-27(1)
O(4)	75(1)	56(1)	55(1)	9(1)	39(1)	-15(1)
C(1)	46(2)	67(2)	50(1)	-14(1)	28(1)	-9(1)
C(2)	38(1)	53(1)	34(1)	-15(1)	19(1)	-17(1)
C(3)	46(1)	49(1)	34(1)	-13(1)	24(1)	-23(1)
C(4)	53(2)	41(1)	38(1)	-7(1)	25(1)	-21(1)
C(5)	49(1)	36(1)	36(1)	-8(1)	24(1)	-14(1)
C(9)	66(2)	36(2)	49(2)	0	34(2)	0
C(7)	41(1)	54(1)	25(1)	-7(1)	16(1)	-15(1)
C(6)	40(1)	44(1)	31(1)	-12(1)	18(1)	-16(1)
C(8)	49(1)	59(2)	36(1)	-5(1)	26(1)	-7(1)
C(10)	60(2)	74(2)	39(1)	-11(1)	32(1)	-15(1)

Table 5. Hydrogen coordinates (x 10^4) and isotropic displacement parameters (Å^{2}x 10 ^{3}) for Bis-CoQ$_0$ 1.

	x	y	z	U(eq)
H(1A)	2277	-898	3331	78

H(1B)	2730	-1422	4390	78
H(1C)	2597	1974	3968	78
H(9A)	24	8188	2033	57
H(9B)	-24	8188	2967	57
H(8A)	133	5103	4166	69
H(8B)	615	3158	4966	69
H(8C)	131	1440	4102	69
H(10A)	1681	627	1726	81
H(10B)	1679	3958	1314	81
H(10C)	1148	2740	1350	81

Table 6. Torsion angles [°] for Bis-CoQ$_0$ 1.

C(1)-O(1)-C(2)-C(3)	-2.2(3)
C(1)-O(1)-C(2)-C(7)	177.89(18)
O(1)-C(2)-C(3)-O(3)	-4.3(4)
C(7)-C(2)-C(3)-O(3)	175.54(18)
O(1)-C(2)-C(3)-C(4)	-178.6(2)
C(7)-C(2)-C(3)-C(4)	1.3(3)
C(10)-O(3)-C(3)-C(2)	114.8(2)
C(10)-O(3)-C(3)-C(4)	-70.7(3)
C(2)-C(3)-C(4)-O(4)	173.9(2)
O(3)-C(3)-C(4)-O(4)	-0.7(3)
C(2)-C(3)-C(4)-C(5)	-4.2(3)
O(3)-C(3)-C(4)-C(5)	-178.79(17)
O(4)-C(4)-C(5)-C(6)	-171.4(2)
C(3)-C(4)-C(5)-C(6)	6.6(3)
O(4)-C(4)-C(5)-C(9)	3.1(3)
C(3)-C(4)-C(5)-C(9)	-178.83(18)
C(6)-C(5)-C(9)-C(5)#1	-71.87(19)
C(4)-C(5)-C(9)-C(5)#1	113.91(18)
O(1)-C(2)-C(7)-O(2)	-1.4(3)
C(3)-C(2)-C(7)-O(2)	178.7(2)
O(1)-C(2)-C(7)-C(6)	179.19(18)
C(3)-C(2)-C(7)-C(6)	-0.7(3)
C(4)-C(5)-C(6)-C(7)	-6.1(3)
C(9)-C(5)-C(6)-C(7)	179.90(19)
C(4)-C(5)-C(6)-C(8)	173.60(19)
C(9)-C(5)-C(6)-C(8)	-0.4(3)

O(2)-C(7)-C(6)-C(5)	-176.1(2)
C(2)-C(7)-C(6)-C(5)	3.3(3)
O(2)-C(7)-C(6)-C(8)	4.2(3)
C(2)-C(7)-C(6)-C(8)	-176.44(19)

Symmetry transformations used to generate equivalent atoms:

#1 -x,y,-z+1/2

Table 7. Hydrogen bonds for Bis-CoQ$_0$ 1 [Å and °].

D-H...A	d(D-H)	d(H...A)	d(D...A)	<(DHA)
C(1)-H(1B)...O(1)#2	0.98	2.60	3.428(4)	142.6
C(10)-H(10C)...O(4)	0.98	2.50	2.990(4)	110.6

Symmetry transformations used to generate equivalent atoms:

#1 -x,y,-z+1/2 #2 -x+1/2,-y-1/2,-z+1

Single Crystal Data of Bis-CoQ$_0$ 2.

Table 1. Crystal data and structure refinement for Bis-CoQ$_0$ 2.

Identification code	mo_dm13388_0m	
Empirical formula	C24 H22 O8	
Formula weight	438.41	
Temperature	140(2) K	
Wavelength	0.71073 Å	
Crystal system	Tetragonal	
Space group	P 43 21 2	
Unit cell dimensions	a = 7.7412(14) Å	α= 90 °.
	b = 7.7412(14) Å	β= 90 °.
	c = 35.232(6) Å	γ = 90 °.
Volume	2111.3(9) Å^3	
Z	4	
Density (calculated)	1.379 Mg/m^3	
Absorption coefficient	0.104 mm^{-1}	
F(000)	920	
Crystal size	0.200 x 0.190 x 0.030 mm^3	
Theta range for data collection	2.312 to 30.563 °.	
Index ranges	-11<=h<=11, -11<=k<=9, -45<=l<=50	
Reflections collected	20875	
Independent reflections	3223 [R(int) = 0.0548]	
Completeness to theta = 25.242 °	100.0 %	
Absorption correction	Semi-empirical from equivalents	
Max. and min. transmission	0.7461 and 0.6486	
Refinement method	Full-matrix least-squares on F^2	
Data / restraints / parameters	3223 / 0 / 148	
Goodness-of-fit on F^2	1.037	
Final R indices [I>2sigma(I)]	R1 = 0.0598, wR2 = 0.1560	
R indices (all data)	R1 = 0.0802, wR2 = 0.1713	
Absolute structure parameter	1.0(5)	
Extinction coefficient	n/a	
Largest diff. peak and hole	0.533 and -0.194 e.Å^{-3}	

Table 2. Atomic coordinates (x 10^4) and equivalent isotropic displacement parameters (Å^{2}x 10^3) for Bis-CoQ$_0$ 2. U(eq) is defined as one third of the trace of the orthogonalized U^{ij} tensor.

	x	y	z	U(eq)
O(1)	5881(3)	2773(4)	8040(1)	48(1)

O(2)	8004(3)	1796(4)	8568(1)	50(1)
O(3)	2206(3)	4689(3)	9043(1)	46(1)
O(4)	2749(3)	4552(3)	8275(1)	45(1)
C(1)	5366(4)	3075(4)	8402(1)	34(1)
C(2)	3888(4)	3820(4)	8520(1)	33(1)
C(3)	3551(4)	4031(4)	8934(1)	31(1)
C(4)	4920(4)	3530(3)	9209(1)	27(1)
C(5)	6441(4)	2896(4)	9088(1)	31(1)
C(6)	6699(4)	2534(4)	8675(1)	34(1)
C(7)	4611(6)	2409(6)	7756(1)	60(1)
C(8)	7956(5)	2457(5)	9334(1)	45(1)
C(9)	4547(4)	3887(4)	9617(1)	26(1)
C(10)	3271(4)	2966(4)	9809(1)	32(1)
C(11)	5490(4)	5148(4)	9809(1)	28(1)
C(12)	1037(5)	3825(5)	8273(1)	51(1)

Table 3. Bond lengths [Å] and angles [°] for Bis-CoQ$_0$ 2.

O(1)-C(1)	1.359(3)
O(1)-C(7)	1.429(4)
O(2)-C(6)	1.221(4)
O(3)-C(3)	1.221(4)
O(4)-C(2)	1.358(4)
O(4)-C(12)	1.440(4)
C(1)-C(2)	1.347(5)
C(1)-C(6)	1.471(5)
C(2)-C(3)	1.493(4)
C(3)-C(4)	1.486(4)
C(4)-C(5)	1.345(4)
C(4)-C(9)	1.491(3)
C(5)-C(6)	1.493(4)
C(5)-C(8)	1.499(4)
C(7)-H(7A)	0.9800
C(7)-H(7B)	0.9800
C(7)-H(7C)	0.9800
C(8)-H(8A)	0.9800
C(8)-H(8B)	0.9800
C(8)-H(8C)	0.9800
C(9)-C(10)	1.393(4)

C(9)-C(11)	1.394(4)
C(10)-C(10)#1	1.390(5)
C(10)-H(10)	0.9500
C(11)-C(11)#1	1.397(5)
C(11)-H(11)	0.9500
C(12)-H(12A)	0.9800
C(12)-H(12B)	0.9800
C(12)-H(12C)	0.9800

C(1)-O(1)-C(7)	119.3(3)
C(2)-O(4)-C(12)	115.9(3)
C(2)-C(1)-O(1)	127.7(3)
C(2)-C(1)-C(6)	121.1(2)
O(1)-C(1)-C(6)	111.1(3)
C(1)-C(2)-O(4)	122.4(3)
C(1)-C(2)-C(3)	119.7(3)
O(4)-C(2)-C(3)	117.6(3)
O(3)-C(3)-C(4)	120.8(2)
O(3)-C(3)-C(2)	120.1(3)
C(4)-C(3)-C(2)	118.9(3)
C(5)-C(4)-C(3)	120.8(2)
C(5)-C(4)-C(9)	122.9(2)
C(3)-C(4)-C(9)	116.1(2)
C(4)-C(5)-C(6)	119.7(3)
C(4)-C(5)-C(8)	125.7(2)
C(6)-C(5)-C(8)	114.6(2)
O(2)-C(6)-C(1)	120.7(3)
O(2)-C(6)-C(5)	120.1(3)
C(1)-C(6)-C(5)	119.2(3)
O(1)-C(7)-H(7A)	109.5
O(1)-C(7)-H(7B)	109.5
H(7A)-C(7)-H(7B)	109.5
O(1)-C(7)-H(7C)	109.5
H(7A)-C(7)-H(7C)	109.5
H(7B)-C(7)-H(7C)	109.5
C(5)-C(8)-H(8A)	109.5
C(5)-C(8)-H(8B)	109.5
H(8A)-C(8)-H(8B)	109.5
C(5)-C(8)-H(8C)	109.5

H(8A)-C(8)-H(8C)	109.5
H(8B)-C(8)-H(8C)	109.5
C(10)-C(9)-C(11)	119.6(2)
C(10)-C(9)-C(4)	120.7(2)
C(11)-C(9)-C(4)	119.7(2)
C(10)#1-C(10)-C(9)	120.28(15)
C(10)#1-C(10)-H(10)	119.9
C(9)-C(10)-H(10)	119.9
C(9)-C(11)-C(11)#1	120.09(14)
C(9)-C(11)-H(11)	120.0
C(11)#1-C(11)-H(11)	120.0
O(4)-C(12)-H(12A)	109.5
O(4)-C(12)-H(12B)	109.5
H(12A)-C(12)-H(12B)	109.5
O(4)-C(12)-H(12C)	109.5
H(12A)-C(12)-H(12C)	109.5
H(12B)-C(12)-H(12C)	109.5

Symmetry transformations used to generate equivalent atoms:

#1 y,x,-z+2

Table 4. Anisotropic displacement parameters ($Å^2 x 10^3$) for Bis-CoQ_0 2. The anisotropic displacement factor exponent takes the form: $-2\pi^2[h^2 a^{*2}U^{11} + ... + 2 h k a^* b^* U^{12}]$

	U^{11}	U^{22}	U^{33}	U^{23}	U^{13}	U^{12}
O(1)	60(2)	66(2)	19(1)	-6(1)	8(1)	-9(1)
O(2)	53(2)	62(2)	36(1)	-10(1)	12(1)	13(1)
O(3)	47(1)	56(1)	36(1)	-9(1)	-7(1)	16(1)
O(4)	58(2)	42(1)	35(1)	6(1)	-15(1)	-3(1)
C(1)	50(2)	34(1)	18(1)	-3(1)	7(1)	-9(1)
C(2)	49(2)	28(1)	23(1)	2(1)	-5(1)	-7(1)
C(3)	42(2)	28(1)	23(1)	-4(1)	0(1)	1(1)
C(4)	38(1)	25(1)	19(1)	-2(1)	4(1)	0(1)
C(5)	42(2)	30(1)	21(1)	-2(1)	7(1)	1(1)
C(6)	46(2)	31(1)	25(1)	-3(1)	9(1)	-2(1)
C(7)	68(2)	88(3)	24(1)	-15(2)	2(2)	-15(2)
C(8)	48(2)	57(2)	31(1)	-2(1)	2(1)	19(2)
C(9)	34(1)	29(1)	16(1)	-2(1)	2(1)	4(1)
C(10)	39(2)	34(1)	23(1)	-6(1)	4(1)	-5(1)

C(11)	32(1)	31(1)	22(1)	0(1)	4(1)	-1(1)
C(12)	46(2)	46(2)	61(2)	-5(2)	-16(2)	4(2)

Table 5. Hydrogen coordinates (x 10^4) and isotropic displacement parameters (Å^{2}x 10 ^{3}) for Bis-CoQ_0 2.

	x	y	z	U(eq)
H(7A)	3727	1642	7863	90
H(7B)	5164	1844	7539	90
H(7C)	4072	3489	7673	90
H(8A)	7645	2627	9601	68
H(8B)	8932	3208	9270	68
H(8C)	8283	1248	9293	68
H(10)	2606	2125	9677	38
H(11)	6366	5775	9680	34
H(12A)	1114	2563	8289	77
H(12B)	445	4150	8038	77
H(12C)	386	4266	8491	77

Table 6. Torsion angles [°] for Bis-CoQ_0 2.

C(7)-O(1)-C(1)-C(2)	-32.1(5)
C(7)-O(1)-C(1)-C(6)	150.2(3)
O(1)-C(1)-C(2)-O(4)	-7.1(5)
C(6)-C(1)-C(2)-O(4)	170.4(3)
O(1)-C(1)-C(2)-C(3)	179.7(3)
C(6)-C(1)-C(2)-C(3)	-2.8(4)
C(12)-O(4)-C(2)-C(1)	120.0(3)
C(12)-O(4)-C(2)-C(3)	-66.6(4)
C(1)-C(2)-C(3)-O(3)	-179.3(3)
O(4)-C(2)-C(3)-O(3)	7.2(4)
C(1)-C(2)-C(3)-C(4)	4.5(4)
O(4)-C(2)-C(3)-C(4)	-169.1(2)
O(3)-C(3)-C(4)-C(5)	-176.2(3)
C(2)-C(3)-C(4)-C(5)	0.0(4)
O(3)-C(3)-C(4)-C(9)	-0.2(4)
C(2)-C(3)-C(4)-C(9)	175.9(2)
C(3)-C(4)-C(5)-C(6)	-5.8(4)

C(9)-C(4)-C(5)-C(6)	178.5(3)
C(3)-C(4)-C(5)-C(8)	175.8(3)
C(9)-C(4)-C(5)-C(8)	0.1(5)
C(2)-C(1)-C(6)-O(2)	177.0(3)
O(1)-C(1)-C(6)-O(2)	-5.1(4)
C(2)-C(1)-C(6)-C(5)	-3.0(4)
O(1)-C(1)-C(6)-C(5)	174.9(3)
C(4)-C(5)-C(6)-O(2)	-172.5(3)
C(8)-C(5)-C(6)-O(2)	6.1(4)
C(4)-C(5)-C(6)-C(1)	7.5(4)
C(8)-C(5)-C(6)-C(1)	-173.9(3)
C(5)-C(4)-C(9)-C(10)	-115.5(3)
C(3)-C(4)-C(9)-C(10)	68.7(3)
C(5)-C(4)-C(9)-C(11)	64.1(4)
C(3)-C(4)-C(9)-C(11)	-111.7(3)
C(11)-C(9)-C(10)-C(10)#1	-1.5(5)
C(4)-C(9)-C(10)-C(10)#1	178.1(3)
C(10)-C(9)-C(11)-C(11)#1	-0.4(5)
C(4)-C(9)-C(11)-C(11)#1	180.0(3)

Symmetry transformations used to generate equivalent atoms:

#1 y,x,-z+2

Table 7. Hydrogen bonds for Bis-CoQ$_0$ 2 [Å and °].

D-H...A	d(D-H)	d(H...A)	d(D...A)	<(DHA)
C(12)-H(12A)...O(3)#2	0.98	2.64	3.538(5)	151.8
C(12)-H(12C)...O(3)	0.98	2.42	2.938(4)	112.2

Symmetry transformations used to generate equivalent atoms:

#1 y,x,-z+2 #2 -x+1/2,y-1/2,-z+7/4

Single Crystal Data of Bis-CoQ$_0$ 3.

Table 1. Crystal data and structure refinement for Bis-CoQ$_0$ 3.

Identification code	DM13455	
Empirical formula	C30 H26 O8	
Formula weight	514.51	
Temperature	296(2) K	
Wavelength	1.54178 Å	
Crystal system	Monoclinic	
Space group	P 2/c	
Unit cell dimensions	a = 8.9316(9) Å	α= 90 °.
	b = 5.7682(8) Å	β= 90.199(11) °.
	c = 24.102(4) Å	γ = 90 °.
Volume	1241.7(3) Å^3	
Z	2	
Density (calculated)	1.376 Mg/m^3	
Absorption coefficient	0.829 mm^{-1}	
F(000)	540	
Crystal size	0.150 x 0.050 x 0.020 mm^3	
Theta range for data collection	3.668 to 69.137 °.	
Index ranges	-10<=h<=9, -6<=k<=6, -28<=l<=28	
Reflections collected	10191	
Independent reflections	2219 [R(int) = 0.1764]	
Completeness to theta = 67.679 °	97.1 %	
Absorption correction	Semi-empirical from equivalents	
Max. and min. transmission	0.7532 and 0.5238	
Refinement method	Full-matrix least-squares on F^2	
Data / restraints / parameters	2219 / 0 / 175	
Goodness-of-fit on F^2	1.121	
Final R indices [I>2sigma(I)]	R1 = 0.0682, wR2 = 0.1691	
R indices (all data)	R1 = 0.1301, wR2 = 0.2417	
Extinction coefficient	n/a	
Largest diff. peak and hole	0.478 and -0.421 e.Å^{-3}	

Table 2. Atomic coordinates (x 10^4) and equivalent isotropic displacement parameters (Å^{2}x 10^3) for Bis-CoQ$_0$ 3. U(eq) is defined as one third of the trace of the orthogonalized U^{ij} tensor.

	x	y	z	U(eq)
O(1)	6316(2)	3314(4)	4101(1)	58(1)
O(2)	1588(2)	8668(4)	3863(1)	57(1)

O(3)	4000(2)	2161(5)	4671(1)	70(1)
O(4)	1273(2)	4944(5)	4535(1)	60(1)
C(1)	9282(3)	6892(5)	2660(1)	36(1)
C(2)	8322(3)	4995(5)	2611(2)	44(1)
C(3)	7012(3)	4896(5)	2916(2)	45(1)
C(4)	6633(3)	6659(5)	3279(1)	37(1)
C(5)	7572(3)	8573(5)	3323(1)	38(1)
C(6)	8888(3)	8679(5)	3011(1)	38(1)
C(7)	5228(3)	6432(5)	3615(1)	36(1)
C(8)	5214(3)	4521(5)	4026(2)	42(1)
C(9)	3807(3)	3987(6)	4342(2)	44(1)
C(10)	2582(3)	5303(6)	4257(1)	45(1)
C(11)	2651(3)	7343(5)	3896(1)	40(1)
C(12)	4027(3)	7821(5)	3560(1)	38(1)
C(13)	3902(3)	9798(6)	3168(2)	47(1)
C(14)	2888(4)	1356(7)	5045(2)	61(1)
C(15)	55(3)	4137(8)	4193(2)	66(1)

Table 3. Bond lengths [Å] and angles [°] for Bis-CoQ$_0$ 3.

O(1)-C(8)	1.219(3)
O(2)-C(11)	1.221(3)
O(3)-C(9)	1.330(4)
O(3)-C(14)	1.421(4)
O(4)-C(10)	1.365(4)
O(4)-C(15)	1.439(5)
C(1)-C(6)	1.379(4)
C(1)-C(2)	1.395(4)
C(1)-C(1)#1	1.500(5)
C(2)-C(3)	1.385(4)
C(2)-H(2)	0.9300
C(3)-C(4)	1.385(4)
C(3)-H(3)	0.9300
C(4)-C(5)	1.390(4)
C(4)-C(7)	1.502(4)
C(5)-C(6)	1.399(4)
C(5)-H(5)	0.9300
C(6)-H(6)	0.9300
C(7)-C(12)	1.345(4)

C(7)-C(8)	1.482(4)
C(8)-C(9)	1.504(4)
C(9)-C(10)	1.346(4)
C(10)-C(11)	1.464(4)
C(11)-C(12)	1.501(4)
C(12)-C(13)	1.484(4)
C(13)-H(13A)	0.9600
C(13)-H(13B)	0.9600
C(13)-H(13C)	0.9600
C(14)-H(14A)	0.9600
C(14)-H(14B)	0.9600
C(14)-H(14C)	0.9600
C(15)-H(15A)	0.9600
C(15)-H(15B)	0.9600
C(15)-H(15C)	0.9600

C(9)-O(3)-C(14)	123.2(3)
C(10)-O(4)-C(15)	114.6(3)
C(6)-C(1)-C(2)	118.7(3)
C(6)-C(1)-C(1)#1	122.38(19)
C(2)-C(1)-C(1)#1	118.9(2)
C(3)-C(2)-C(1)	120.5(3)
C(3)-C(2)-H(2)	119.7
C(1)-C(2)-H(2)	119.7
C(2)-C(3)-C(4)	120.9(3)
C(2)-C(3)-H(3)	119.5
C(4)-C(3)-H(3)	119.5
C(3)-C(4)-C(5)	118.8(3)
C(3)-C(4)-C(7)	118.9(2)
C(5)-C(4)-C(7)	122.3(3)
C(4)-C(5)-C(6)	120.2(3)
C(4)-C(5)-H(5)	119.9
C(6)-C(5)-H(5)	119.9
C(1)-C(6)-C(5)	120.9(3)
C(1)-C(6)-H(6)	119.6
C(5)-C(6)-H(6)	119.6
C(12)-C(7)-C(8)	120.1(3)
C(12)-C(7)-C(4)	124.3(3)
C(8)-C(7)-C(4)	115.7(2)

O(1)-C(8)-C(7)	121.0(3)
O(1)-C(8)-C(9)	119.0(3)
C(7)-C(8)-C(9)	120.0(2)
O(3)-C(9)-C(10)	129.9(3)
O(3)-C(9)-C(8)	110.9(2)
C(10)-C(9)-C(8)	119.2(3)
C(9)-C(10)-O(4)	122.5(3)
C(9)-C(10)-C(11)	120.5(3)
O(4)-C(10)-C(11)	116.8(3)
O(2)-C(11)-C(10)	120.6(3)
O(2)-C(11)-C(12)	119.1(3)
C(10)-C(11)-C(12)	120.3(2)
C(7)-C(12)-C(13)	125.4(3)
C(7)-C(12)-C(11)	119.4(3)
C(13)-C(12)-C(11)	115.1(2)
C(12)-C(13)-H(13A)	109.5
C(12)-C(13)-H(13B)	109.5
H(13A)-C(13)-H(13B)	109.5
C(12)-C(13)-H(13C)	109.5
H(13A)-C(13)-H(13C)	109.5
H(13B)-C(13)-H(13C)	109.5
O(3)-C(14)-H(14A)	109.5
O(3)-C(14)-H(14B)	109.5
H(14A)-C(14)-H(14B)	109.5
O(3)-C(14)-H(14C)	109.5
H(14A)-C(14)-H(14C)	109.5
H(14B)-C(14)-H(14C)	109.5
O(4)-C(15)-H(15A)	109.5
O(4)-C(15)-H(15B)	109.5
H(15A)-C(15)-H(15B)	109.5
O(4)-C(15)-H(15C)	109.5
H(15A)-C(15)-H(15C)	109.5
H(15B)-C(15)-H(15C)	109.5

Symmetry transformations used to generate equivalent atoms:

#1 -x+2,y,-z+1/2

Table 4. Anisotropic displacement parameters ($Å^2$x 10^3) for Bis-CoQ_0 3. The anisotropic displacement factor exponent takes the form: $-2\pi^2[h^2 a^{*2}U^{11} + ... + 2 h k a^* b^* U^{12}]$

	U^{11}	U^{22}	U^{33}	U^{23}	U^{13}	U^{12}
O(1)	39(1)	63(2)	74(2)	22(1)	14(1)	16(1)
O(2)	40(1)	61(2)	70(2)	4(1)	9(1)	18(1)
O(3)	47(1)	83(2)	80(2)	43(2)	23(1)	15(1)
O(4)	35(1)	92(2)	53(2)	15(1)	15(1)	7(1)
C(1)	30(1)	33(2)	44(2)	2(1)	7(1)	2(1)
C(2)	38(1)	35(2)	58(2)	-13(2)	16(1)	-3(1)
C(3)	38(1)	39(2)	58(2)	-7(2)	16(2)	-7(1)
C(4)	30(1)	38(2)	43(2)	5(1)	7(1)	2(1)
C(5)	32(1)	37(2)	46(2)	-5(1)	6(1)	4(1)
C(6)	32(1)	34(2)	49(2)	0(1)	2(1)	-2(1)
C(7)	31(1)	37(2)	41(2)	0(1)	7(1)	2(1)
C(8)	30(1)	45(2)	51(2)	5(2)	5(1)	5(1)
C(9)	36(1)	52(2)	44(2)	12(2)	8(1)	6(1)
C(10)	30(1)	65(2)	40(2)	3(2)	11(1)	3(1)
C(11)	28(1)	49(2)	44(2)	-3(1)	0(1)	6(1)
C(12)	32(1)	42(2)	39(2)	-2(1)	4(1)	1(1)
C(13)	41(1)	46(2)	55(2)	6(2)	0(2)	3(1)
C(14)	61(2)	71(2)	52(2)	22(2)	16(2)	-2(2)
C(15)	36(1)	80(3)	82(3)	12(2)	6(2)	0(1)

Table 5. Hydrogen coordinates (x 10^4) and isotropic displacement parameters ($Å^2$x 10 ^{3}) for Bis-CoQ_0 3.

	x	y	z	U(eq)
H(2)	8564	3786	2372	52
H(3)	6377	3629	2876	54
H(5)	7325	9785	3560	46
H(6)	9505	9972	3040	46
H(13A)	4874	10165	3024	71
H(13B)	3505	11122	3359	71
H(13C)	3245	9385	2868	71
H(14A)	2624	2580	5296	92
H(14B)	3272	62	5252	92
H(14C)	2017	882	4840	92
H(15A)	-251	5351	3945	99
H(15B)	-772	3705	4425	99

H(15C)	376	2817	3982	99

Table 6. Torsion angles [°] for Bis-CoQ$_0$ 3.

C(6)-C(1)-C(2)-C(3)	-1.0(5)
C(1)#1-C(1)-C(2)-C(3)	177.6(3)
C(1)-C(2)-C(3)-C(4)	-0.8(5)
C(2)-C(3)-C(4)-C(5)	1.9(5)
C(2)-C(3)-C(4)-C(7)	-177.9(3)
C(3)-C(4)-C(5)-C(6)	-1.2(4)
C(7)-C(4)-C(5)-C(6)	178.5(3)
C(2)-C(1)-C(6)-C(5)	1.6(4)
C(1)#1-C(1)-C(6)-C(5)	-177.0(3)
C(4)-C(5)-C(6)-C(1)	-0.5(4)
C(3)-C(4)-C(7)-C(12)	-114.4(3)
C(5)-C(4)-C(7)-C(12)	65.9(4)
C(3)-C(4)-C(7)-C(8)	65.3(4)
C(5)-C(4)-C(7)-C(8)	-114.5(3)
C(12)-C(7)-C(8)-O(1)	-176.4(3)
C(4)-C(7)-C(8)-O(1)	3.9(4)
C(12)-C(7)-C(8)-C(9)	5.9(5)
C(4)-C(7)-C(8)-C(9)	-173.7(3)
C(14)-O(3)-C(9)-C(10)	-5.2(6)
C(14)-O(3)-C(9)-C(8)	175.8(3)
O(1)-C(8)-C(9)-O(3)	-0.4(5)
C(7)-C(8)-C(9)-O(3)	177.3(3)
O(1)-C(8)-C(9)-C(10)	-179.5(3)
C(7)-C(8)-C(9)-C(10)	-1.8(5)
O(3)-C(9)-C(10)-O(4)	2.1(6)
C(8)-C(9)-C(10)-O(4)	-179.0(3)
O(3)-C(9)-C(10)-C(11)	176.2(3)
C(8)-C(9)-C(10)-C(11)	-4.9(5)
C(15)-O(4)-C(10)-C(9)	-112.5(4)
C(15)-O(4)-C(10)-C(11)	73.2(4)
C(9)-C(10)-C(11)-O(2)	-172.6(3)
O(4)-C(10)-C(11)-O(2)	1.8(5)
C(9)-C(10)-C(11)-C(12)	7.8(5)
O(4)-C(10)-C(11)-C(12)	-177.7(3)
C(8)-C(7)-C(12)-C(13)	179.6(3)

C(4)-C(7)-C(12)-C(13)	-0.8(5)
C(8)-C(7)-C(12)-C(11)	-3.1(4)
C(4)-C(7)-C(12)-C(11)	176.6(3)
O(2)-C(11)-C(12)-C(7)	176.8(3)
C(10)-C(11)-C(12)-C(7)	-3.6(4)
O(2)-C(11)-C(12)-C(13)	-5.6(4)
C(10)-C(11)-C(12)-C(13)	174.0(3)

Symmetry transformations used to generate equivalent atoms:

#1 -x+2,y,-z+1/2

Table 7. Hydrogen bonds for Bis-CoQ$_0$ 3 [Å and °].

D-H...A	d(D-H)	d(H...A)	d(D...A)	<(DHA)
C(14)-H(14B)...O(1)#2	0.96	2.52	3.462(4)	166.5
C(15)-H(15A)...O(2)	0.96	2.53	3.058(5)	114.7
C(15)-H(15C)...O(2)#3	0.96	2.64	3.531(5)	154.0

Symmetry transformations used to generate equivalent atoms:

#1 -x+2,y,-z+1/2 #2 -x+1,-y,-z+1 #3 x,y-1,z
